# Mobilization of isotopically heavy sulfur during serpentinite subduction

**DOI:** 10.1126/sciadv.adn0641

**Published:** 2024-08-07

**Authors:** Esther M. Schwarzenbach, Besim Dragovic, Emmanuel A. Codillo, Linus Streicher, Maria Rosa Scicchitano, Uwe Wiechert, Frieder Klein, Horst R. Marschall, Marco Scambelluri

**Affiliations:** ^1^ Institute of Geological Sciences, Freie Universität Berlin, Berlin, Germany.; ^2^ Department of Geosciences, University of Fribourg, Fribourg, Switzerland.; ^3^ School of the Earth, Ocean & Environment, University of South Carolina, Columbia, SC, USA.; ^4^ Massachusetts Institute of Technology-Woods Hole Oceanographic Institution Joint Program in Oceanography/Applied Ocean Science and Engineering, Woods Hole, MA, USA.; ^5^ Earth and Planets Laboratory, Carnegie Institution for Science, Washington DC, USA.; ^6^ Deutsches GeoForschungsZentrum GFZ, Potsdam, Germany.; ^7^ Department of Marine Chemistry and Geochemistry, Woods Hole Oceanographic Institution, Woods Hole, MA, USA.; ^8^ Institut für Geowissenschaften, Goethe Universität, Frankfurt am Main, Germany.; ^9^ Department of Earth, Environmental and Life Sciences, University of Genoa, Genoa, Italy.

## Abstract

Primitive arc magmas are more oxidized and enriched in sulfur-34 (^34^S) compared to mid-ocean ridge basalts. These findings have been linked to the addition of slab-derived volatiles, particularly sulfate, to arc magmas. However, the oxidation state of sulfur in slab fluids and the mechanisms of sulfur transfer in the slab remain inconclusive. Juxtaposed serpentinite and eclogitic metagabbro from the Voltri Massif (Italy) provide evidence for sulfur mobilization and associated redox processes during infiltration of fluids. Using bulk rock and in situ δ^34^S measurements, combined with thermodynamic calculations, we document the transfer of bisulfide-dominated, ^34^S-enriched fluids in equilibrium with serpentinite into adjacent metagabbro. We argue that the process documented in this study is pervasive along the subduction interface and infer that subsequent melting of these reacted slab-mantle interface rocks could produce melts that display the characteristic oxygen fugacity and sulfur isotope signatures of arc magmas worldwide.

## Teaser

Reactive flow at the slab-mantle interface of fluids derived from hydrated mantle explains the sulfur signatures of arc magmas.

## INTRODUCTION

The delivery and transport of sulfur into Earth’s interior in subduction zones fundamentally affects Earth’s long-term chemical and redox evolution, the formation of arc magmas, and associated economically important ore deposits [e.g., (*1*–*10*)]. Subducting oceanic plates contain sulfur in altered oceanic crust (*11*, *12*), serpentinized lithospheric mantle (*13*), and sediments (*14*). This transport process from the subducting oceanic slab to the subarc mantle, feeding arc magmas, has been suggested to explain the elevated sulfur contents of many primitive arc magmas relative to mid-ocean ridge basalt (MORB) (*15*, *16*). In particular, the release and transport of ^34^S-enriched sulfate, which is initially derived from seawater, during slab dehydration or melting, or both, have been linked to the ^34^S-enriched S compositions and the higher oxidation states of primitive arc magmas worldwide relative to MORB (*6*, *15*, *17*–*22*). Delivery of oxidized slab components is thought to be essential for controlling oxygen fugacity (*f*o_2_) in subduction zones, and sulfate-bearing fluids, in particular, have been proposed as an ideal candidate for an oxidizing agent, with the capability to oxidize 8 mol of Fe(II) (in mantle minerals) to Fe(III) by reducing only 1 mol of SO_4_^2−^ to S^2−^ [e.g., (*1*, *6*)].

The mobilization and release of sulfur as sulfate during subduction are evident at sulfate-rich serpentinite mud volcanoes in the Mariana and Izu-Bonin forearcs (*23*). Its presence in subduction zones has also been inferred from stable isotope measurements of zinc and sulfur in exhumed serpentinite-dominated mélange rocks (*7*, *24*, *25*). Furthermore, positive δ^34^S values and high U/Th (as well as Pb/Ce and Sr/Nd) ratios measured in subarc-derived mantle xenoliths and melt inclusions in Cascadia and Kamchatka arcs (*16*, *26*) were taken to suggest the involvement of subducted serpentinite as the main carrier of large amounts of ^34^S-enriched oxidized S to subarc mantle depths (*2*, *26*–*28*). However, the redox state of redox-sensitive elements in fluids released by serpentinite dehydration is still controversial, and whether these fluids act as reducing (*29*, *30*) or oxidizing agents (*26*, *27*) upon reaction with surrounding rocks remains to be unraveled.

Hence, despite evidence for the transport of ^34^S-enriched S from the subducting slab into the source of arc magmas, there is no consensus on the exact origin and redox state of ^34^S-enriched S and on the nature of its transfer into the subarc mantle and into the source region of arc magmas. This partly stems from the lack of a mechanistic understanding of the behavior of sulfur and its impact on S isotope fractionation at the slab-mantle interface in subduction zones. For instance, Li *et al.* (*3*) highlight a discrepancy between the average δ^34^S signature of slab dehydration fluids released at subarc depths [δ^34^S = −2.5 ± 3 per mil (‰)] and the positive δ^34^S values of arc magmas. In contrast, Li *et al.* (*31*) and Walters *et al.* (*7*) provide evidence for substantial S isotope variability of slab fluids as a result of the presubduction geodynamic and hydrothermal history, with an estimate on slab fluids covering a large range from −11 to +8‰ in δ^34^S (*7*). Alternatively, it has been suggested that the fairly uniform positive δ^34^S values in arc lavas may be the result of assimilation of seawater-altered crust in the upper plate (*32*). Moreover, metasomatism and associated fluid migration within the slab may cause changes in the *f*o_2_ of the fluid as shown for exhumed metamorphosed samples from Greece, where it is suggested that slab dehydration fluids can increase their *f*o_2_ by passage through metasediments during their ascent to the mantle wedge (*33*, *34*). These processes can have a large impact on δ^34^S signatures due to substantial isotope fractionation between relatively more oxidized (e.g., SO_4_^2−^ and S^6+^) and more reduced (e.g., H_2_S, HS^−^, and S^2−^) sulfur species and could further modify the δ^34^S signatures of slab fluids.

To better understand the role and importance of slab-derived sulfur in subduction zones, it is critical to determine the mechanisms of sulfur transformation and transport during metasomatic reactions that are shown to be pervasive within the slab and along the slab-mantle interface. Here, we investigate sulfur speciation during fluid-mediated mass transfer between serpentinite and sulfide-bearing eclogitic metagabbros from the high-pressure Voltri Massif (Ligurian Alps, Italy). We build upon the previous petrological findings by Codillo *et al.* (*35*) who reported fluid-mediated mass transfer of Mg between serpentinite and metagabbro that initiated during burial and continued through peak and slightly postpeak *P*-*T* conditions. We present mineralogical, geochemical, isotopic, and thermodynamic data that are consistent with the mobilization and transfer of ^34^S-enriched sulfur as HS^−^-dominated fluids from serpentinite into the metagabbro during subduction.

## GEOLOGICAL SETTING

**Fig. 1.**
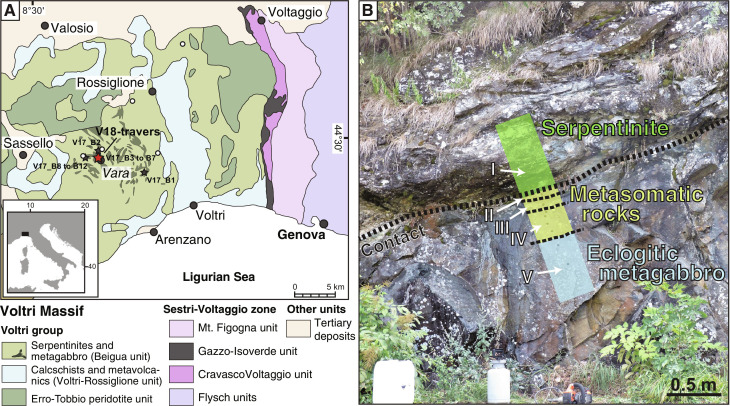
Geological setting and sampling location in the Voltri Massif. (**A**) Geological map of the Voltri Massif (Italy) with the sampling locations of the studied samples marked as stars. Metagabbro bodies are indicated as dark green areas. Map after Vignaroli *et al.* (*91*) and Starr *et al.* (*40*). White circles represent sampling locations of serpentinites presented in Alt *et al.* (*51*) and Schwarzenbach (*52*). (**B**) Outcrop image showing the serpentinite-metagabbro transect [red star in (A)] with the location of the lithological zones I to V.

The Voltri Massif is part of the Ligurian Alps in Italy, at the eastern end of the Western Alps, bordering the transition to the northern portion of the Apennine Mountains. Exposed lithologies represent ophiolitic sections that were formed as part of the oceanic lithosphere in the Late Jurassic Liguro-Piedmont Ocean with the protoliths of the Voltri Massif being mantle peridotites, Fe-Ti–rich gabbroic intrusions, MORB, and oceanic sediments (*36*–*39*). Subduction-related metamorphism in the central portion of the Voltri Massif reached eclogite-facies conditions with maximum *P*-*T* conditions of 500° to 525°C and 2.3 to 2.5 GPa at ~38 to 40 Ma (*39*, *40*). On the basis of relatively uniform *P*-*T*-*t* histories of the Fe-Ti gabbros, it is concluded that exhumation took place as >5-km-large coherent sections of oceanic lithosphere, at least for the central part of the Voltri Massif (*35*, *40*), the exhumation of individual blocks within a serpentinite mélange being confined to localized areas in the massif (*41*). The investigated rock samples are from the Voltri Massif and were collected near the village of Vara (Fig. 1A). They are primarily eclogitic metagabbros that experienced variable degrees of metasomatism and retrograde overprinting during exhumation (*35*, *42*). We collected samples from different locations in the western Voltri Massif, where metagabbros occur as meter-scale blocks, as well as along a ~3.5-m transect across a serpentinite-metagabbro contact, where eclogitic metagabbros were increasingly metasomatized through fluid-mediated mass transfer by a serpentinite-equilibrated Mg-rich fluid resulting in complete conversion to chlorite-bearing actinolite schists along the immediate contact (Fig. 1B) (*35*). Serpentinite was collected within 2 m from the contact, and variably metasomatized eclogitic metagabbros were collected within 1.5 m from the contact, with two additional metagabbros sampled from the same eclogite body at ~10 and ~20 m from the contact.

## RESULTS

### Mineralogical description

The studied samples along the 3.5-m transect include antigorite serpentinite and variably metasomatized eclogitic Fe-Ti metagabbro, which have previously been grouped into five different zones according to their mineralogical composition and distance to the serpentinite-metagabbro contact (Fig. 1B) (*35*): Zone I samples are antigorite serpentinite from within 2 m of the contact, zone II (~0 to 0.02 m from the contact) is composed of chlorite-bearing actinolite schists (<5% chlorite) and was not studied here, zone III consists of actinolite-chlorite schists (~0.02 to 0.2 m), zone IVa consists of epidote- and Na-Ca-amphibole–rich metagabbros (~0.2 to 0.35 m), zone IVb are composed of Na-Ca-amphibole–rich metagabbros (~0.35 to 1.5 m), and zone V are the eclogitic Fe-Ti metagabbros (sampled at ~10 and ~20 m from the contact) that were not affected by Mg metasomatism. The eclogitic Fe-Ti metagabbros from throughout the Beigua unit generally experienced variable degrees of deformation and, thus, have previously been grouped into massive or “coronitic” Fe-Ti metagabbro and foliated or “mylonitic” Fe-Ti metagabbro (*39*). The coronitic metagabbros still preserve igneous textures with garnet coronae around omphacitic pyroxene, whereas the mylonitic metagabbros do not preserve igneous textures. Instead, they consist of euhedral garnet surrounded by a foliated matrix (*40*). For a detailed description of the chemical and mineralogical compositions of each of the zones and lithologies, we refer to Messiga and Scambelluri (*39*), Starr *et al.* (*40*), and Codillo *et al.* (*35*).

**Fig. 2.**
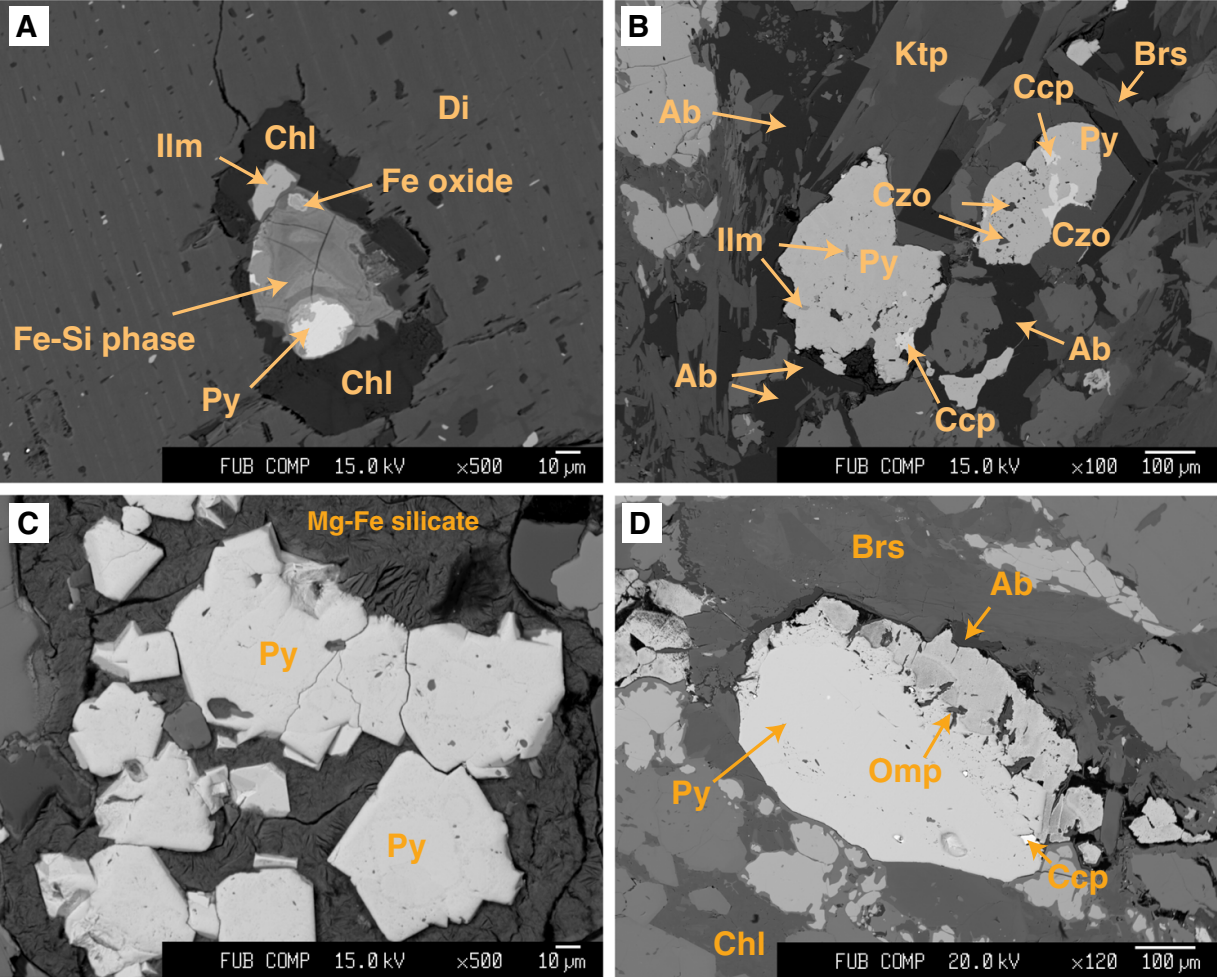
Mineralogical and petrological overview of the sulfide minerals. Backscattered electron images of sulfides in the studied metagabbros. (**A**) Pyrite grain in an actinolite-chlorite schist (zone III) within diopside. Pyrite is strongly corroded to Fe oxide and a hydrous Fe-Si phase, and chlorite is surrounding it. (**B**) Pyrite intergrown with chalcopyrite and containing silicate and oxide inclusions (zone IVa). (**C**) Euhedral pyrite grains in a fibrous Mg-Fe silicate (zone IVa). (**D**) Pyrite with corrosion rim and silicate inclusions (zone IVb).

**Fig. 3.**
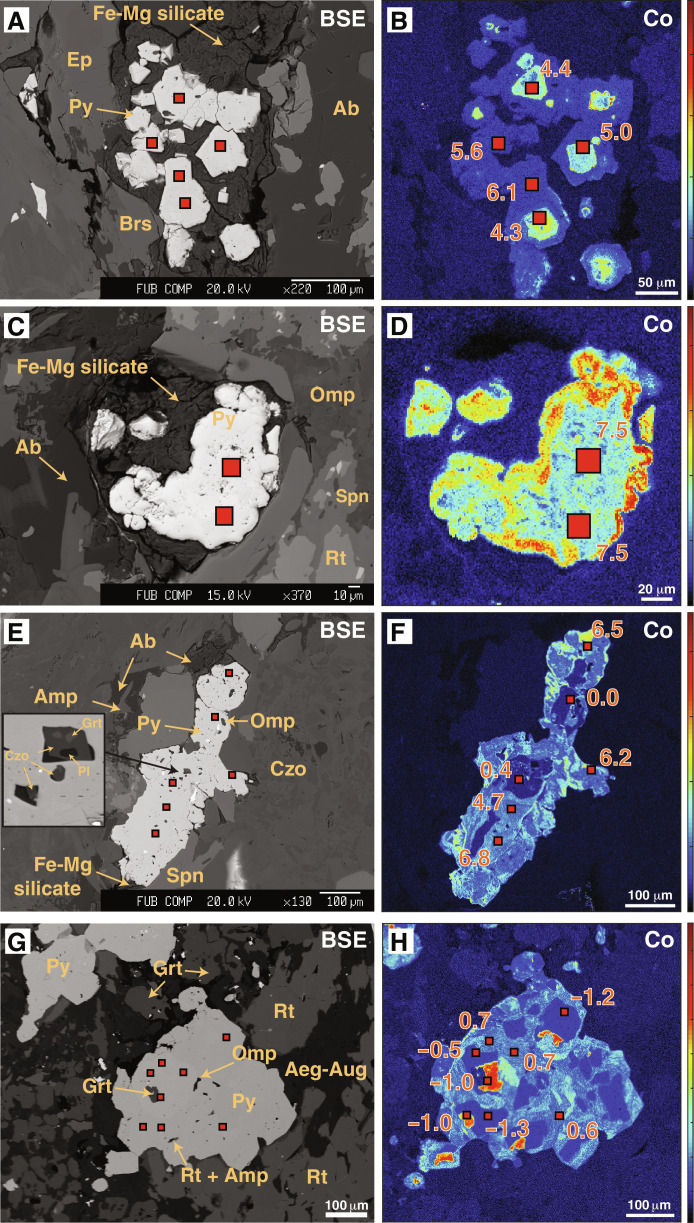
In situ sulfur isotope analyses of selected pyrite grains. Secondary ion mass spectrometry (SIMS) spots are shown as red squares in the backscattered electron (BSE) images (left column) and in the Co element distribution maps (right column). (**A** and **B**) Pyrite in zone IVa, (**C** to **F**) pyrite in zone IVb, and (**G** and **H**) pyrite in zone V. Note that, in (E) and (F), metamorphic minerals including Omp, Grt, and Pl occur in inner zones that are rimmed by pyrite with high δ^34^S values (4.7 to 6.8‰). δ^34^S values are expressed as per mil versus Vienna Canyon Diablo Troilite (V-CDT) in the Co maps. In the element maps, red colors represent high concentrations, while cold (to black) colors represent low concentrations.

Our focus is on the sulfide minerals that, with increasing distance from the serpentinite-metagabbro contact, occur in different generations. In the investigated serpentinite, no sulfide or sulfate minerals were detected either by microscopic observations or by chemical extractions. In the actinolite-chlorite schist (zone III), sulfides are rare and occur as grains of <50 μm in diameter. These sulfide grains are mostly pyrite, which are always highly corroded and rimmed by a chemically zoned, hydrous Fe-Si phase (Fig. 2A), and euhedral pyrrhotite and chalcopyrite grains occur rimmed by chlorite, included within diopside porphyroblasts. With further distance from the contact, pyrite is the dominant sulfide in all samples. In zone IVa, it occurs either as highly corroded pyrite that is intergrown with chalcopyrite or trace amounts of covellite (CuS), bornite (Cu_5_FeS_4_), and idaite (Cu_5_FeS_6_) (Fig. 2B and fig. S1) or as euhedral pyrite (Fig. 2C) associated with albite, calcic amphibole, or a fibrous Mg-Fe silicate that has a chemical composition between greenalite and talc (but could not be determined further). In many cases, euhedral pyrite grains show distinct zonation with respect to Co, with a Co-rich core (up to 1.6 wt % of Co) and a Co-poor rim (<0.2 wt % of Co) (Fig. 3, A and B). In these pyrite grains, silicate mineral inclusions are rare. In some pyrite overgrowth rims, Co zonation is patchy or oscillatory (fig. S2), with the rims typically having Co contents of <1 wt %. In these pyrite grains, mineral silicate inclusions consist of omphacite, ilmenite, albite, epidote, and Na-Ca amphiboles (Fig. 2D). In zone IVb, pyrite occurrences are the same as in zone IVa, but pyrite grains have a Co-poorer core and a Co-enriched rim (Fig. 3, C to F). The Co-poor core contains inclusions of omphacite, amphiboles (barroisite and katophorite), and epidote, and, in one grain, we observed garnet + plagioclase rimmed by epidote (see Fig. 3E). Most of these mineral phases are also found as inclusions in the Co-enriched rim.

In zone V, the eclogitic metagabbro, pyrite is subhedral to anhedral. Subhedral pyrite shows distinct growth zones of variable Co and Ni contents (see Fig. 3H and fig. S3), with the innermost zone (i) having Co contents up to 4.5 wt % but very low Ni contents (<0.02 wt %), followed by a zone (ii) of very low Co (<0.16 wt %) and elevated Ni (0.07–0.73 wt %), and an outer zone (iii) with intermediate Co (0.64 to 1.10 wt %) and low Ni contents (<0.02 wt %). In most grains, the innermost zone contains no inclusions, whereas the outer two zones contain some to abundant inclusions of omphacite, garnet, epidote, amphiboles (winchite, barroisite, and katophorite), rutile, and rare quartz and albite. Among others, the eclogite-facies mineral assemblage omphacite + garnet + quartz is present in the outermost zone. In addition, note that none of the studied samples contain sulfide grains included within garnet.

### Sulfur contents and isotope compositions

**Fig. 4.**
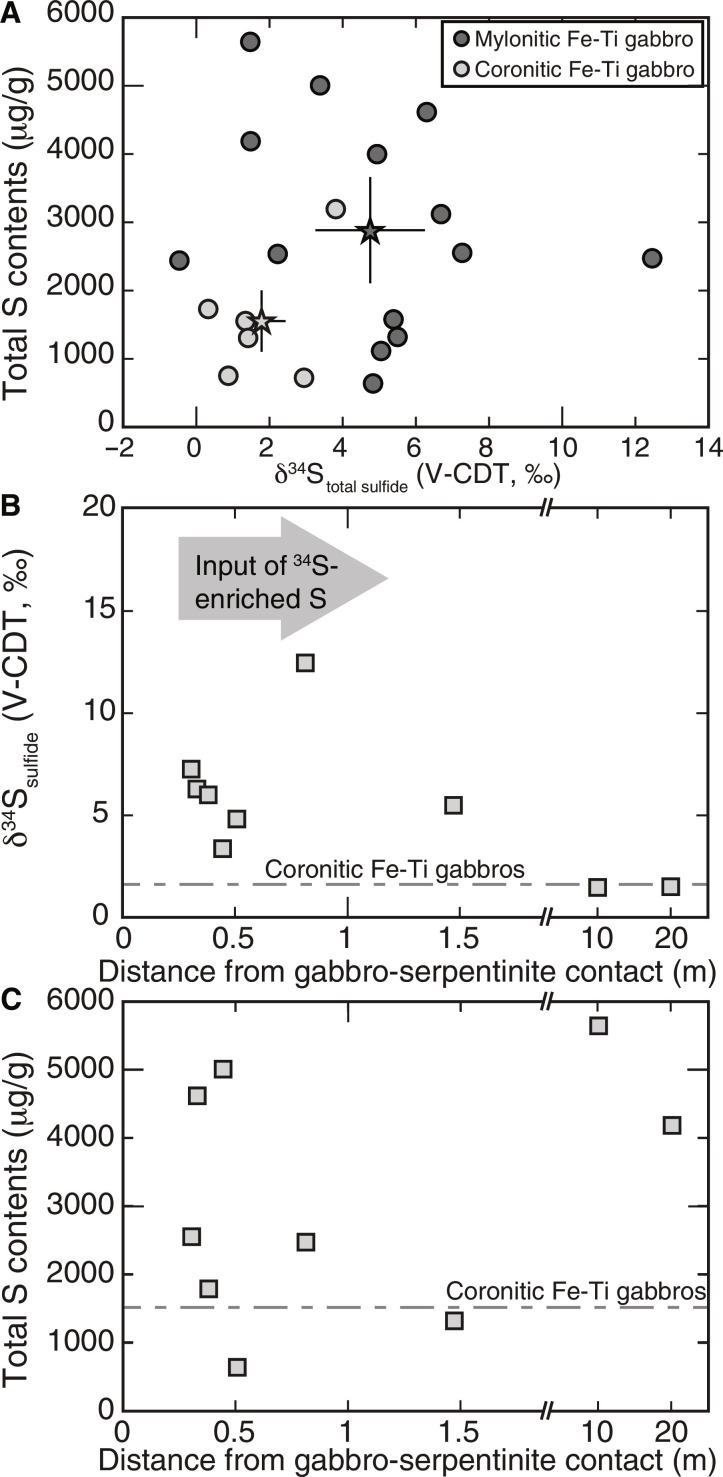
Bulk rock S contents and isotope compositions. (**A**) Mylonitic Fe-Ti metagabbros have, on average, (black star) higher bulk rock total sulfide contents compared to average coronitic (gray star) metagabbros. (**B**) δ^34^S_sulfide_ values and (**C**) total sulfide contents of samples collected along the serpentinite-metagabbro contact shown with distance to the contact indicating input of ^34^S-enriched S associated with metasomatism. Average S compositions of all coronitic Fe-Ti gabbros (*n* = 6) is shown as gray dashed line in (B) and (C).

Bulk rock sulfur contents determined by wet chemical sulfur extraction are highly variable in the studied samples, particularly along the serpentinite-metagabbro transect (Fig. 4 and data S1). Serpentinite (zone I) and actinolite-chlorite schist (zone III) have sulfide and sulfate contents below detection limit except for one actinolite-chlorite schist that yields sulfate contents of 6 μg/g. This agrees with microscopic observations that only trace amounts of sulfide phases could be detected within 0.3 m from the contact. Farther away from the contact, i.e., in all zones further into the metagabbro sequence, sulfur is highly dominated by sulfide (sulfide/Σ_sulfur_ > 94%) with sulfate contents of <64 μg/g. In zone IV, total sulfide (and total S) contents are highest at 0.3 to 0.4 m from the contact (~1780 to 5000 μg/g) and generally lower between 0.5 and 1.5 m from the contact (~630 to 2470 μg/g) (Fig. 4C), while bulk rock δ^34^S_sulfide_ values in this zone vary between +3.4 and +12.5‰ (Fig. 4B). The two samples of zone V at ~10 and ~20 m from the contact have a bulk rock S and sulfide content of ~4180 to 5640 μg/g and δ^34^S_sulfide_ values of +1.5‰. This is within the range of metagabbro sampled throughout the Voltri Massif with bulk rock sulfide and total S contents of ~710 to 4060 μg/g and δ^34^S_sulfide_ values of −0.5 to +6.7‰. Notably, mylonitic metagabbro has, on average, higher total sulfur contents (2695 ± 1634 μg/g) and more positive δ^34^S_sulfide_ values (δ^34^S_sulfide_ = +4.7 ± 3.2‰) than massive metagabbro (1537 ± 908 μg/g; δ^34^S_sulfide_ = +1.8 ± 1.3‰) (Fig. 4A), while sulfate was only detected in the mylonitic metagabbro with δ^34^S_sulfate_ values of −1.7 to +4.4‰.

**Fig. 5.**
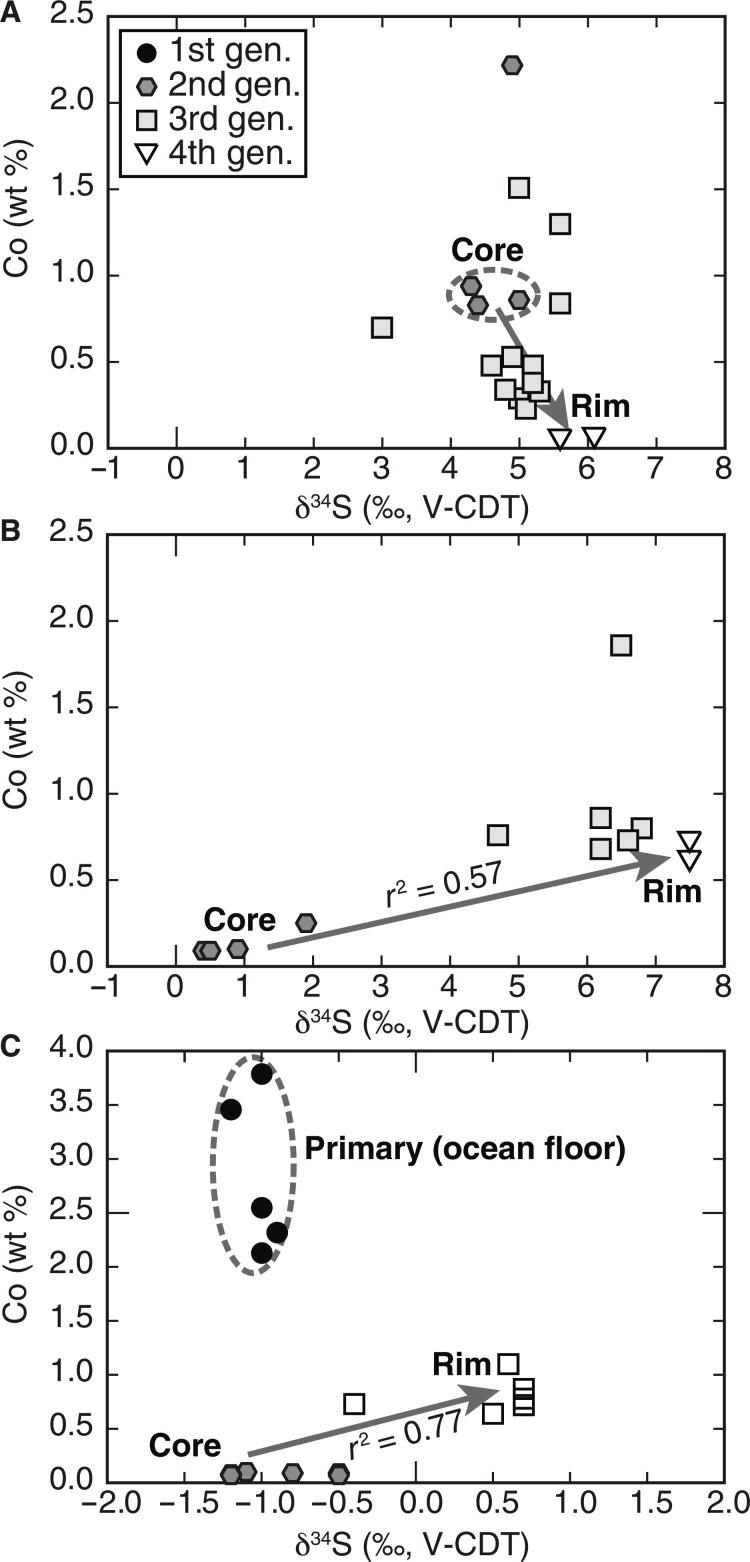
S isotope variation shown by mineral chemistry. Co contents versus in situ δ^34^S values in pyrite grains for zones IVa (**A**), IVb (**B**), and V (**C**). All grains show an increase in δ^34^S values from core to rim. Co zonations are attributed to different growth generations and color-coded from black, gray to white (core to mantle to rim, respectively). Distinct correlations between δ^34^S values and Co content are seen in two samples (*r*^2^ = 0.57 to 0.77). Note that Co compositions represent an approximate value for respective in situ δ^34^S values, since SIMS spots are considerably larger (~10 μm in diameter) than electron microprobe spots.

In situ δ^34^S measurements determined by secondary ion mass spectrometry (SIMS) of pyrite in four metagabbro samples from zones IVa, IVb, and V yielded δ^34^S_sulfide_ values of −1.4 to +7.5‰ (data S2). In two samples, distinct correlations between δ^34^S_sulfide_ values and Co contents are observed (*r*^2^ = 0.56 to 0.77; Fig. 5), whereas the bulk of data generally shows an increase in δ^34^S_sulfide_ values from core to rim of individual pyrite grains correlating with different Co zones as observed in the mappings (fig. S1). In zone IVa, Co-richer cores have δ^34^S values of +4.3 to +5.0‰, and Co-poorer rims have δ^34^S values of +5.6 to +6.1‰ (Figs. 3B and 5A); where Co variations are patchy, in situ δ^34^S values have a narrow range of +4.6 to +5.6‰. In zone IVb, pyrites have Co-poor cores with δ^34^S values of −0.4 to +2.7‰, and Co-richer overgrowth rims with δ^34^S values up to +6.8‰ (Fig. 3F), whereby highest δ^34^S values up to +7.5‰ are measured in euhedral to subhedral pyrite grains that are surrounded by a fibrous Mg-Fe silicate (Figs. 3, C and D, and 5B). In zone V, a narrow δ^34^S range of −1.4 to +0.7‰ is observed. However, associated with Co and Ni variations, three generations of pyrite are evident in zone V samples: (i) a high-Co generation (up to 4 wt %) that has δ^34^S values of −1.2 to −0.9‰, (ii) a low-Co and elevated Ni generation with δ^34^S values of −1.3 to −0.4‰, and (iii) an intermediate Co generation that has higher δ^34^S values of +0.6 to +0.7‰ (Figs. 3H and 5C).

## DISCUSSION

### Primary sulfide composition and the oceanic hydrothermal signature

All subducted slab components that have interacted with the hydrosphere before subduction (sediments, altered oceanic crust, and serpentinized lithospheric mantle) are important hosts of variable amounts of oxidized (e.g., SO_4_^2−^ and S^6+^) and reduced (e.g., S^2−^ and S^−^) sulfur of highly variable isotopic composition [e.g., (*3*, *7*, *31*)]. Ocean floor serpentinites typically contain several thousands of sulfide (in micrograms per gram) with δ^34^S_sulfide_ values of −45 to +27‰ (*13*), fresh gabbro contains sulfide between 100 and 1800 μg/g (*43*), and hydrothermally altered gabbro contains sulfide up to 8500 μg/g (δ^34^S_sulfide_ = −1.5 to +16‰) (*44*) [see Li *et al.* (*3*), Walters *et al.* (*7*), and Alt (*11*) for compilations of δ^34^S signatures for average oceanic lithosphere]. During subduction, additional processes such as sediment compaction and metamorphic dehydration reactions can mobilize and redistribute sulfur via processes such as sulfide dissolution and reprecipitation. Moreover, sulfide-to-sulfate or sulfate-to-sulfide transformations can take place among different subducted rocks and metasomatic minerals newly formed by fluid-rock interactions (*2*, *8*, *25*).

The sulfides observed in the Voltri Massif record distinct generations of sulfide precipitation and S mobilization during their evolution. The Voltri ophiolite suite formed within the slow to ultraslow spreading Liguro-Piemont ocean basin (*38*, *45*, *46*). In this basin, discrete gabbro bodies intrude mantle peridotites, whereas basaltic lava extrusions only discontinuously covered the oceanic basement (*47*). More commonly, tectonic-dominated extension during opening of an ocean basin led to widespread mantle exposure on the ocean floor, inducing serpentinization, and localized exposure of gabbroic bodies to the seafloor (*48*, *49*). Ocean floor alteration signatures are only partly retained in the studied serpentinite samples and are primarily documented by their distinct trace element patterns (*35*). However, the measured bulk rock S geochemistry, particularly the very low sulfide contents, is distinctly different from typical ocean floor serpentinites (*13*, *50*) or serpentinites collected elsewhere in the Voltri Massif (*51*, *52*) (see sampling location in Fig. 1), which contain up to 0.25 wt % of reduced S with δ^34^S_sulfide_ values up to +14.3‰ attributed to ocean floor processes. Similarly, the ^87^Sr/^86^Sr ratios of the studied serpentinite do not record Jurassic seawater but rather fluid input from either a crustal- or a sediment-derived fluid that overprinted previous interaction with Jurassic seawater upon seafloor exposure (*35*, *53*).

The metagabbro similarly retains only a faint chemical record of ocean floor alteration and preserves ^87^Sr/^86^Sr ratios that are close to those of unaltered MORB (*35*). Although the bulk rock δ^34^S_sulfide_ values overlap with those found, for instance, in altered gabbro from Atlantis Bank along the Southwest Indian Ridge (δ^34^S_sulfide_ = −1.5 to +16.3‰) (*44*), the S variability of mylonitic metagabbro can at least partially be related to later-stage fluid-rock interaction as discussed below. Only the massive (coronitic) metagabbro, with average δ^34^S_sulfide,WR_ values of +1.7 ± 1.3‰, provides evidence for magmatic sulfur (δ^34^S_MORB_ ≈ −0.91 ± 0.50‰) (*54*) or sulfide formation during minor ocean floor alteration—e.g., during high-*T* hydrothermal alteration, as documented by a slight shift to higher δ^34^S_sulfide,WR_ values commonly up to ~+5‰ [e.g., (*55*)]. The magmatic origin for some of the sulfide grains is also revealed by Co-poor pyrite cores of zone IVb samples, which have δ^34^S_pyrite_ values ranging from −0.5 to +1‰, and by Co-rich, (silicate) inclusion-free pyrite cores in zone V metagabbro characterized by δ^34^S_pyrite_ values ranging from −1.2 to −0.8‰ (Figs. 3 and 5), which overlap with sulfide compositions in ocean floor gabbros (*56*). An ocean floor origin of pyrite from these zones is further supported by a lack of high-pressure mineral inclusions, which is in contrast to other growth generations (Fig. 3). These magmatic-derived sulfide cores were likely retained as encapsulated relicts within later-stage sulfide growth generations.

### Subduction zone S isotopic imprint

**Fig. 6.**
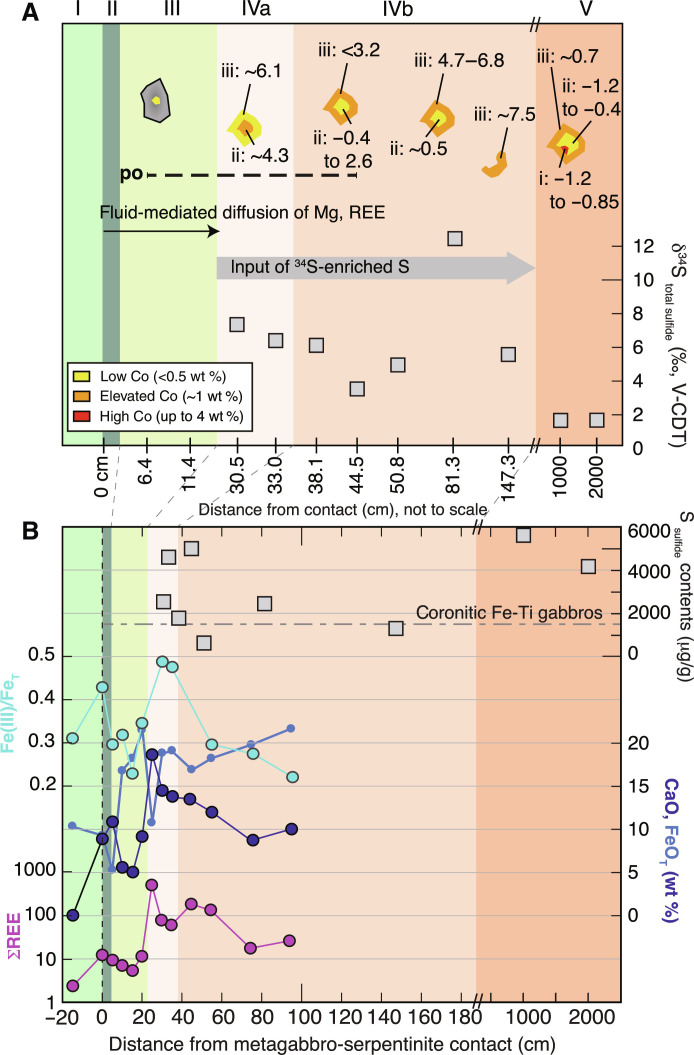
Isotopic and chemical along transect variation. (**A**) Schematic evolution of δ^34^S values in pyrite grains along the serpentinite-metagabbro contact [zones after Codillo *et al.* (*35*)], with the studied samples shown by distance to the contact along the *x* axis (not to scale). Pyrite formation stages are indicated in yellow and orange (growth zones labeled i to iii are shown according to their Co content) with corresponding in situ δ^34^S values. Dashed line labeled “po” indicates presence of pyrrhotite. Also shown are bulk rock δ^34^S_sulfide_ values (gray squares). (**B**) Comparison of bulk rock sulfide, CaO, FeO, and ∑REE (rare earth element) contents and Fe(III)/Fe_total_ ratios with distance from the contact reflecting redox processes associated with metasomatism. CaO, ∑REE, and Fe data are from Codillo *et al.* (*35*).

Previous studies have shown that sulfide minerals are reactive to fluid-rock interaction processes and document relatively short-lived processes taking place during prograde, peak, and retrograde conditions (*3*, *7*, *25*, *57*–*60*). In the studied samples, these processes are preserved by distinct variations of in situ δ^34^S_pyrite_ values, Co contents, and assemblages of silicate mineral inclusions in pyrite, which indicate S mobility during subduction metamorphism. In particular, the presence of peak metamorphic minerals (e.g., omphacite, garnet, and rutile in pyrite cores; Fig. 3E) in the studied samples and mineral assemblages related to reaction-zone formation (Ca-Na amphiboles, epidote, sphene, and ilmenite; Fig. 2) documents that S was mobile during metasomatic processes at conditions prevailing at the slab-mantle interface. Further evidence for S transfer from the serpentinite into the metagabbro during Mg metasomatism is provided by the presence of euhedral to subhedral pyrite that shows distinct core-to-rim variation in the in situ δ^34^S_pyrite_ values. These pyrite grains occur in all samples at distances of 0.3 to 1.5 m from the contact but are absent at larger distance, strongly suggesting that they were associated with the formation of the actinolite and actinolite-chlorite schists along the contact. The observed positive correlation between δ^34^S values and Co contents in two samples (Fig. 5, B and C) and the general increase in the in situ δ^34^S values from core to rim to up to +7.5‰ in samples near the contact that coincides with very sharp Co zonations further points to the influx of ^34^S-enriched sulfur, followed by pyrite (re)crystallization. These observations further coincide with the bulk rock δ^34^S_sulfide_ values being generally higher (+3.4 to +12.5‰) in samples <1.5 m from the contact, compared to +1.1 to +1.9‰ for samples 10 to 20 m from the contact (Fig. 6A) that were less affected by the infiltrating fluid. The lack of a pronounced correlation between bulk rock S contents and δ^34^S values along the serpentinite-metagabbro transect (Fig. 4B) may reflect heterogeneities in S contents and/or δ^34^S values in the protolith (*44*), sample heterogeneity on the centimeter (i.e., sampling) scale, or that fluid influx took place along rather distinct zones. The variation in the Co concentrations can be related to changes in the fluid chemistry, such as the salinity (*59*) and by silicate- and oxide-forming reactions, effectively by changes between fluid- and rock-buffered regimes (*61*). Hence, the observed Co variations likely preserve distinct pyrite forming events on the sample scale during the formation of the metasomatic zone rather than allow inter sample comparison.

Codillo *et al.* (*35*) proposed a model in which the lithologies along the studied contact were modified by fluids that initially equilibrated with serpentinite and subsequently caused mass transfer by fluid-mediated diffusion between serpentinite and metagabbro near peak metamorphic conditions (*35*). This mass transfer was driven by chemical potential gradients, notably those of Mg, Ca, and Si. In addition, the sulfide mineral stabilities reflect the sulfur fugacity (*f*s_2_) and *f*o_2_ and are also controlled by temperature and pressure and by the silicate mineral assemblages, particularly the stability of Fe-bearing mineral phases [e.g., (*2*, *57*, *62*)].

**Fig. 7.**
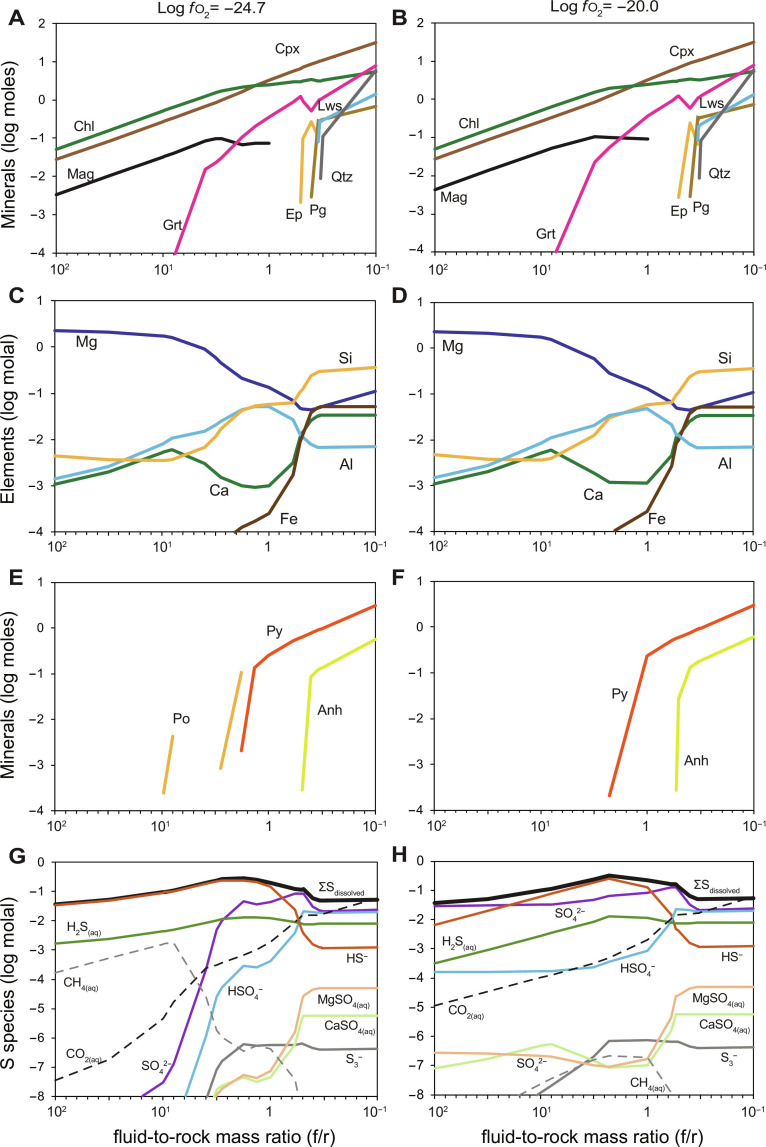
Thermodynamic modeling predictions of mineralogical variations. Predicted silicate and sulfide alteration mineralogy and coexisting fluid composition during high *P*-*T* metasomatism as a function of *f*/*r* mass ratio for (**A**, **C**, **E**, **G**) relatively low *f*o_2_ [log *f*o_2_ = −24.7 or Δlog_10_*f*(O_2_)HM = −6.4] and (**B**, **D**, **F**, **H**) relatively high *f*o_2_ [log *f*o_2_ = −20 or Δlog_10_*f*(O_2_)HM = −1.7]. A fluid equilibrated with serpentinite (at *f*/*r* > 100) is subsequently allowed to react with sulfide-bearing metagabbro at 500°C and 1.5 GPa. The *f*/*r* mass ratio decreases as metagabbro is titrated into the serpentinite-equilibrated fluid. Mineral abbreviations are from Whitney and Evans (*92*).

**Fig. 8.**
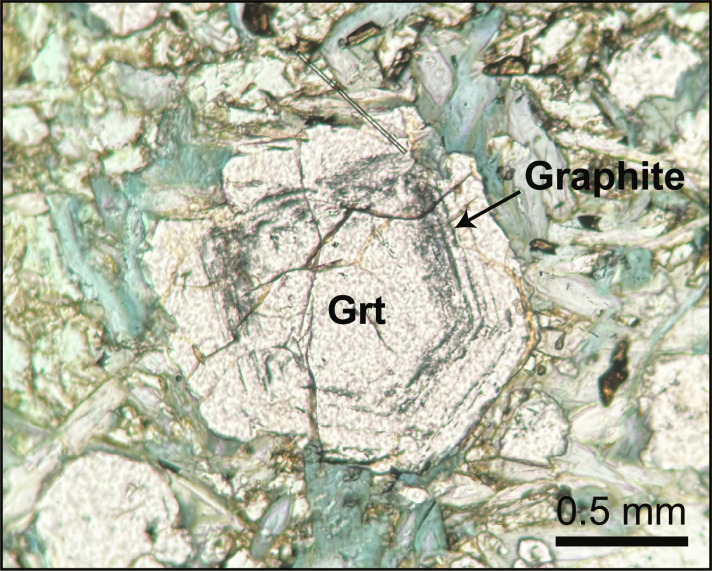
Microphotograph of graphite included in garnet. Graphite forms dark gray to black concentric inclusions in garnet in a metagabbro taken 20 cm from the serpentinite-metagabbro contact.

Thermodynamic reaction path models were constructed (Fig. 7) to further assess the changes in sulfide mineralogy and fluid composition during the fluid-mediated reaction between serpentinite and sulfide-bearing eclogitic metagabbro. In these models, the reactant sulfide-bearing eclogitic metagabbro also contains C (100 μg/g) as a conservative estimate for the carbon content of the rock. This is consistent with petrographic observations of graphite now included within primary silicate minerals (e.g., garnet; Fig. 8). Models were calculated at 500°C and 1.5 GPa over a range of *f*o_2_ where magnetite is thermodynamically stable (bounded by the wüstite-magnetite and hematite-magnetite buffers) and a range of fluid-to-rock (*f*/*r*) mass ratios, using EQ3/6 (*63*) and the Deep Earth Water (DEW) Model database (*64*, *65*) (Fig. 7). Details on the model setup and assumptions are described in Materials and Methods. Metasomatism of pyrite-bearing metagabbro by a fluid previously in equilibrium with serpentinite (antigorite + diopside + magnetite) predicts a sequence of alteration assemblages composed of chlorite + clinopyroxene + garnet + lawsonite + quartz → chlorite + clinopyroxene + epidote ± paragonite → garnet + chlorite + clinopyroxene → clinopyroxene + chlorite + magnetite → clinopyroxene + magnetite + chlorite with increasing *f*/*r* ratios. This alteration sequence broadly reproduces the sequence of metasomatic silicate assemblages observed in this study [see also (*35*)] and suggests that the observed zones represent interaction with fluid at increasing *f*/*r* ratios from metagabbro toward the serpentinite contact. While changes in the starting *f*o_2_ do not affect the calculated silicate mineralogy, the calculated sequence of sulfide assemblages differs over the modeled *f*o_2_ conditions (Fig. 7). Metasomatism of sulfide-bearing metagabbro by serpentinite-buffered fluid at relatively low *f*o_2_ conditions (*f*o_2_ = −24.7) is predicted to form pyrite + anhydrite → pyrite → pyrrhotite with increasing *f*/*r* ratios as the metagabbro is reacting with the fluid. In contrast, pyrrhotite is not predicted in the models that assume higher *f*o_2_ (Fig. 7F). The low-*f*o_2_ model predicts HS^−^ as the dominant sulfur species, whereas the high-*f*o_2_ model predicts HS^−^ and SO_4_^2−^ as the dominant sulfur species in the coexisting fluid at high *f*/*r* ratios. Both models predict increasing concentration of SO_4_^2−^ until it dominates at lower *f*/*r* ratios (<1) (Fig. 7, G and H). With regard to carbon species, CH_4(aq)_ dominates at high *f*/*r* ratios, whereas CO_2(aq)_ dominates at low *f*/*r* ratios, although in the model calculated at higher *f*o_2_, CH_4(aq)_ concentrations are overall very low (Fig. 7H). In both models, anhydrite was predicted at low *f*/*r* ratios. However, the absence of anhydrite in reacted metagabbro could suggest that either it dissolved during sample preparation due to its soluble nature or the models overpredict anhydrite saturation because of the current lack of complexes (ligands) in the database that would suppress anhydrite saturation (*66*).

In general, the models predict a decrease in the concentration of dissolved Mg and increases in the concentrations of dissolved Ca, Fe, and Si with decreasing *f*/*r* ratios (Fig. 7, C and D). The highest concentration of dissolved Al is predicted at intermediate *f*/*r* ratios. Although the models successfully reproduced the observed succession of silicate and sulfide mineralogy, the lack of thermodynamic data for solid solutions at elevated pressure, notably Fe(II) and Fe(III) serpentine (i.e., greenalite and cronstedtite) and the andradite component of garnet, introduce a nonnegligible uncertainty on the modeled *f*o_2_ variations. Because the Fe(III)/Fe_T_ of the modeled serpentinite is dictated by magnetite being the only Fe(III)-bearing phase, the predicted *f*o_2_ of the fluid in equilibrium with the magnetite-bearing serpentinite likely differs from the actual *f*o_2_ in equilibrium with high-pressure serpentinite at Voltri (*35*). However, the ability of the models to successfully reproduce the alteration mineralogy and the sequence of mineral parageneses in the studied serpentinite-metagabbro transect strongly suggest that the assumptions built in the models are justified and that the models broadly capture the mass transfer process between the juxtaposed rocks and the continuous reaction of the metagabbro with a serpentinite-equilibrated fluid at increasing *f*/*r* ratios. We anticipate that improvements in thermodynamic databases will improve our ability to model fluid-rock interactions at high *P*-*T* conditions, especially regarding modeled redox-sensitive species.

To further evaluate the predictions of our reaction-path models, we calculated the *f*o_2_ at which the partial pressures of SO_2_ and H_2_S are equal [i.e., 2 H_2_S_(aq)_ + 3 O_2(g)_ = 2 SO_2(aq)_ + 2 H_2_O], as well as for CO_2_ and CH_4_ [i.e., CH_4(aq)_ + 2 O_2(*g*)_ = CO_2(aq)_ + 2 H_2_O], and compared them to the upper limit of *f*o_2_ wherein magnetite can be stable, as bounded by the hematite-magnetite buffer. Our thermodynamic calculations show that the *f*o_2_ of magnetite-hematite buffer is lower than the SO_2_-H_2_S buffer at 500°C and 1.0 to 2.0 GPa (fig. S4). This suggests that reduced sulfur species (H_2_S) would predominate in fluids coexisting with magnetite-bearing serpentinite at the peak *P*-*T* conditions recorded by the Voltri Massif (~500° to 525°C and 2.3 to 2.5 GPa) (*35*, *40*). This result is consistent with previous petrologic studies that inferred relatively reducing (HS^−^ and H_2_S-bearing) fluids derived from subducted serpentinites (*29*, *57*, *67*). Since magnetite is usually more abundant than pyrite in serpentinite, our calculations also imply that more oxidized sulfur species may predominate in fluids derived from the full dehydration of magnetite-bearing serpentinite at the antigorite-out reaction, which occurs at higher temperatures (>660°C) (*68*). This is indicated by the topology of the equilibrium boundaries wherein the *f*o_2_ buffered by SO_2_-H_2_S equilibrium is lower than the hematite-magnetite buffer at temperatures at or above the serpentinite-out reaction at a constant pressure [fig. S4; see (*67*)].

### Variations in sulfide mineralogy and sulfur speciation during metasomatism

The observed sulfide mineralogy across the studied transect and the very low bulk rock S contents (<0.01 wt % of S) in zone III samples (Fig. 6B) can be related to different redox reactions and the destabilization and dissolution of pyrite and pyrrhotite upon reaction with fluids at increasing *f*/*r* ratios. Note that the dissolution of pyrite (i.e., desulfurization) and the local stabilization of pyrrhotite with increasing *f*/*r* ratio were only observed in reaction-path models performed under relatively low starting *f*o_2_ condition, indicating the sensitivity of the reaction to the prescribed *f*o_2_ conditions.

In general, the redox processes occurring during metasomatism are dominated by coupled changes in the valence states of Fe (i.e., being the most abundant multivalent element in the metagabbro), S, and C among mineral and fluid (*1*). In this regard, the model predictions can provide additional constraints on the stability of sulfide minerals and related changes in the speciation of dissolved S compounds across distinct metasomatic zones. Because of the close match between the predicted and observed sulfide mineral assemblages, we focus on the results from the model with initially reducing conditions (log *f*o_2_ of −24.7). In this model, with decreasing *f*/*r* ratios from 10 to 1, the coexisting fluid shows a decrease in the concentrations of HS^−^ and an increase in the concentrations of dissolved oxidized sulfur species (e.g., SO_4_^2−^ and HSO_4_^−^). The increase in oxidized sulfur species is best explained by reaction with Fe(III) in the Fe-Ti metagabbro such as

$${\text{HS}}^{-}+4\;{\text{Fe}}_{2}O_{3}\;\left(\text{in silicates}\right)={{\text{HSO}}_{4}}^{-}+8\;\text{FeO}\;\left(\text{in silicates}\right)$$ (1)

This process would then allow for an increase in the concentrations of dissolved sulfate as the metagabbro is increasingly reacting with the fluid at intermediate *f*/*r* ratios (i.e., *f*/*r* ≈ 1 to 6; representative of the transition between zones III and IV). At higher *f*/*r* ratios of ~10, pyrrhotite is predicted to form, whereas pyrite disappears. The reduction of pyrite to pyrrhotite requires a reducing agent and can be explained by the introduction of CH_4_, which is dominantly stable at high *f*/*r* ratios associated with highly reducing conditions, following the reaction:

$$2\;{\text{FeS}}_{2}+{\text{CH}}_{4}=\text{FeS}+H_{2}S+C$$ (2)

The local presence of graphite as inclusions in garnet (Fig. 8) supports the involvement of C during fluid-rock interaction. Moreover, this coincides with the observation of pyrrhotite in zone III, near the contact with the serpentinite where the infiltrating fluid is dominated by reducing species. Overall, it is likely that Fe, S, and C are involved in the redox reactions taking place during metasomatism and that they can also be coupled through other reaction such as

$${\text{CH}}_{4}+4\;{\text{FeS}}_{2}+6\;H_{2}O=8\;H_{2}S+4\;\text{FeO}+{\text{CO}}_{2}$$ (3)

and

$${\text{CH}}_{4}+2\;{\text{FeS}}_{2}+2\;H_{2}O=4\;H_{2}S+2\;\text{FeO}+C$$ (4)

While the actual amount of carbon originally present in the rock or fluid is unknown, the conservative value initially assumed in the models allow us to explore the role of carbon species in the redox reactions with sulfur and iron. Furthermore, the redox conditions are likely variable between distinct metasomatic zones, which can cause local dissolution of redox sensitive minerals, such as sulfides, in some parts and precipitation in other parts of this transect. This is reflected by the large variations in bulk Fe(III)/Fe_T_ contents and sulfide modal contents observed over short length scales across the different reaction zones. For instance, toward the interior of the metagabbro (away from the contact), the variations in bulk rock Fe(III)/Fe_T_ ratios and CaO [as well as ΣREE (rare earth element)] contents in zone IVa coincide with changes in sulfide contents and δ^34^S_sulfide,WR_ values. In particular, the elevated sulfide contents are associated with metasomatic rocks with high bulk rock Fe(III)/Fe_T_ ratios, CaO and ΣREE contents, and an abundance of epidote minerals (Fig. 6B). Codillo *et al.* (*35*) performed mass-balance calculations and argued that the formation of epidote-rich zone IVa required the addition of Ca from the adjacent zone III. In Fig. 7, the thermodynamic models suggest that at an *f*/*r* ratio of ~1, the predicted fluid composition displays increasing concentrations in dissolved Ca and SO_4_^2−^, wherein the latter dominates the dissolved sulfur budget (ΣS_dissolved_). The predicted availability of SO_4_^2−^ and Ca in the fluids likely promoted the stabilization of epidote and pyrite in zone IVa. The reduction of dissolved SO_4_^2−^ during pyrite precipitation is most likely coupled to the oxidation of Fe(II) in metagabbro, as evidenced by the locally elevated bulk rock Fe(III)/Fe_T_ in zone IVa, and can be described by the reaction:

$$\begin{array}{c} {{\text{SO}}_{4}}^{2-}+15\;{\text{Fe}}^{2+}O\;\left(\text{in silicates}\right)+2\;{\text{Ca}}^{2+}={\text{FeS}}_{2}\;\left(\text{pyrite}\right)\\ +7\;{{\text{Fe}}^{3+}}_{2}O_{3}\;\left(\text{in epidote}\right)+2\;\text{CaO}\;\left(\text{in epidote}\right) \end{array}$$ (5)

This agrees with previous studies recording the reduction of SO_2_ (S^4+^) or SO_4_^2−^ (S^6+^) as aqueous species associated with the oxidation of surrounding lithologies [e.g., (*7*, *57*, *67*)]. Quantifying the exact sequence of reactions remains challenging because of the uncertainty in the amount of C and S that was initially present in the rock before metasomatism. Nonetheless, we speculate that if the initial metagabbro had the same or much higher carbon content than assumed in our simplified thermodynamic models, then carbon would certainly be important in the redox reactions with sulfur, in addition to Fe.

**Fig. 9.**
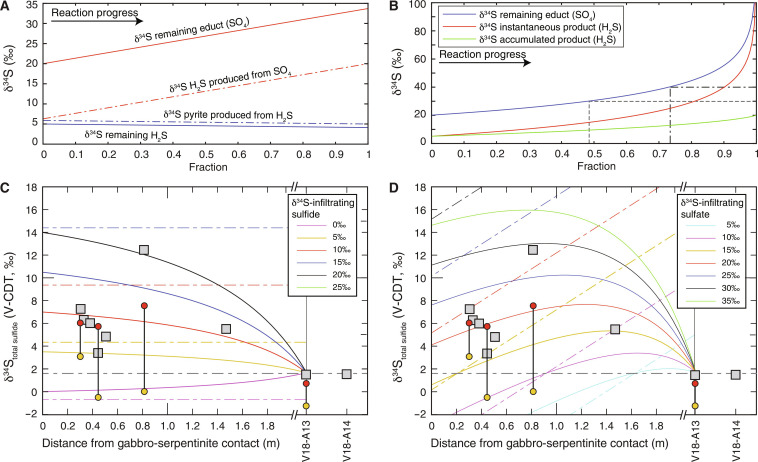
Calculations of δ^34^S compositions during fluid evolution associated with sulfur isotope fractionation and mixing. (**A**) Closed system fluid evolution with sulfate reduction to produce sulfide (red lines) assuming initial δ^34^S_sulfate_ = 20‰, where fluid evolution results in final δ^34^S_H__2__S_ values equal to the initial δ^34^S_sulfate_. Pyrite precipitation from H_2_S (blue lines) assuming δ^34^S_H__2__S_ = 5‰ is associated with minor isotope fractionation. (**B**) Rayleigh distillation model assuming an initial δ^34^S sulfate value of 20‰ whereby δ^34^S values of the produced H_2_S continuously increase during SO_4_^2−^ reduction to H_2_S. (**C**) Mixing model of gabbro with incremental addition of H_2_S with different δ^34^S values derived from the serpentinite. Pyrite H_2_S fractionation with ε_pyrite-__H__2__S_ = 0.9‰ at 400°C (*90*) is assumed. Solid lines represent bulk rock compositions produced by mixing, and colored dashed lines represent respective δ^34^S values of the introduced H_2_S. (**D**) Mixing model of gabbro with incremental addition of sulfide produced by closed system sulfate reduction [see (A)], whereby the δ^34^S_H__2__S_ produced from sulfate reduction continuously increases (colored dashed lines). Solid lines represent bulk rock compositions produced by mixing. In (C) and (D), the gabbro has a composition of sulfide (1500 μg/g) and δ^34^S_sulfide_ = 1.7‰ (gray dashed line), and the end member metagabbro unaffected by metasomatism along the traverse is set to a hypothetical point at ~2-m distance from the contact. For comparison, the measurement of sample V18-A13 is shown at this position. Red- and orange-colored circles represent rim and core compositions, respectively, of in situ δ^34^S measurements in pyrite. For all models, *T* = 400°C.

On the basis of the in situ δ^34^S_pyrite_ measurements it can be concluded that the S isotope composition of the infiltrating, subduction-related fluid did not change substantially as it was introduced into the metagabbro, since the measured maximum in situ δ^34^S values of sulfide rims are around +6‰ at ~0.3 m and +7.5‰ at ~0.8 m from the contact. Within the subducting slab and particularly in the case of the Voltri Massif, the two most dominant sources of ^34^S-enriched S include (i) seawater-derived sulfate primarily stored within sediments or serpentinites (as S^6+^ or SO_4_^2−^) and (ii) ^34^S-enriched sulfide minerals from serpentinized peridotites that were formed during serpentinization and hydrothermal alteration (*13*, *69*). In particular, oceanic peridotites that undergo serpentinization at low *f*/*r* ratios and temperatures above ~300°C can have δ^34^S values of up to +23.1‰ but typically in the range of +2 to +14‰ (*69*). These positive δ^34^S values are similar to values measured in serpentinites from the Beigua unit, with sampling localities very close to those measured in this study (see Fig. 1A), that are mostly +6.9 to +14.3‰ (*51*). Thus, we can assume that the studied serpentinites had similarly positive δ^34^S values before their interaction with a subduction-related fluid. This coincides with many exhumed high-pressure serpentinites that show predominantly positive δ^34^S values (*3*, *16*, *70*). Furthermore, our thermodynamic models predict that sulfide is undersaturated in metagabbro that reacted at very high *f*/*r* ratios with fluids previously equilibrated with serpentinite (Fig. 7, G and H), with limited isotope fractionation expected during sulfide dissolution [e.g., (*71*)]. This means that the resultant dissolved HS^−^ would carry a ^34^S-enriched S signature very similar to the precursor sulfide minerals in serpentinite. As the HS^−^-bearing, ^34^S-enriched fluid starts to react with the metagabbro following Eq. 1, SO_4_^2−^ concentrations initially increase. Subsequently, at decreasing *f*/*r* ratios and as SO_4_^2−^ continuously infiltrates and reacts with the surrounding metagabbro, pyrite precipitates following, e.g., Eq. 5. Reduction and reprecipitation (SO_4_^2−^ ➔ H_2_S ➔ pyrite) as manifested in zone IVa would not have caused any substantial isotope fractionation under closed system fluid evolution (Fig. 9A and see Materials and Methods for details). Rayleigh fractionation processes would have led to highly positive δ^34^S values (Fig. 9B) and, thus, are also unlikely to have been an important process for the investigated metasomatic zone. However, bulk mixing between the infiltrating fluid and the distal metagabbro (δ^34^S_gabbro_ ≈ +1.7‰) could produce a S isotope variation as observed in the investigated samples. The observed trend in δ^34^S composition along the transect is best explained by the incremental addition of ^34^S-enriched S to the metagabbro, whereby continuous pyrite precipitates at decreasing *f*/*r* ratios, stripping sulfur out of the infiltrating fluid (shown by decreasing HS^−^ concentrations at low *f*/*r* ratios; Fig. 7G). This translates to a decreasing influx of ^34^S-enriched S with increasing distance from the contact and that higher δ^34^S values are observed at slightly higher *f*/*r*, i.e., where the S from the infiltrating fluid is dominating. Such a scenario can be well reproduced using initial δ^34^S_sulfide_ values of approximately +5 to +15‰, agreeing with estimates of serpentinized peridotites from the Voltri Massif (Fig. 9C and fig. S5).

Alternatively, the influx of seawater-derived sulfate hosted by oceanic serpentinite could similarly have led to the influx of ^34^S-enriched component. Oceanic serpentinites can contain seawater-derived sulfate of several hundreds of micrograms per gram up to 0.4 wt % (*72*). However, the Sr isotope compositions of the studied serpentinite are more radiogenic than Jurassic seawater and require interaction with fluids derived from continental or sedimentary rocks (*35*, *53*). This process was suggested to have occurred during subduction by migration of fluids derived from subducted materials along the subduction interface (*53*, *73*). On the basis of mixing and isotope fractionation models, sulfate with δ^34^S values of +15 to +22‰ could have similarly led to ^34^S-enriched values in pyrite in the reacted metagabbro following the conversion of SO_4_^2−^ to HS^−^ at 400°C (Fig. 9D). These δ^34^S_sulfate_ values agree with seawater sulfate of Jurassic to modern ages (*74*) and, thus, would coincide with ocean floor alteration and sedimentation in the Liguro-Piemont Ocean. However, considering that oxidized S species (e.g., SO_4_^2−^) were not predicted to be the dominant species in the modeled fluid in equilibrium with magnetite-bearing serpentinite at the *P*-*T* conditions relevant to the Voltri Massif (Fig. 7D), we suggest that this process may be less important. Further evidence that a contact parallel fluid circulation did not directly provide the ^34^S-enriched signature to metagabbro (with sulfate sourced from sediments) is given by the Sr signatures and the trace element patterns provided elsewhere (*35*). If this were the case, then circulation of sediment-derived fluid and sulfur metasomatism of metagabbro would display distinct collateral geochemical changes, most notably on the Sr isotope composition of the metagabbro, as well as additional sediment-derived chemical signatures. However, the constant Sr isotope ratios (0.7037 ± 0.0001) of the metagabbro across the transect argue against direct sulfur addition into the metagabbro by fluids that were sourced and last equilibrated with sedimentary sequences through contact parallel fluid circulation (*35*). Instead, the lack of any substantial Sr isotope variations in the metagabbro that record sulfur metasomatism indicates that the ^34^S-enriched S was most likely derived from the adjacent serpentinite, although the influx from sediment-derived S cannot be excluded entirely. Because serpentinite has very low Sr concentrations (0.77 ± 0.33 μg/g; *n* = 9), serpentinite-buffered fluids are also likely to contain very low concentrations of dissolved Sr. This type of fluid could carry the ^34^S-enriched S from serpentinite without modifying the Sr budget and Sr isotope composition of the metagabbro upon reaction. The higher δ^34^S values in the mylonitic metagabbros compared to the coronitic metagabbros may further suggest that ^34^S-enriched fluids equilibrating with surrounding serpentinites likely affected the rather small metagabbro bodies during deformation on a larger scale. Last, as constrained previously (*35*), the Mg-rich fluid equilibrated with serpentinite at high pressures, and it is constrained that the metasomatic zone formed and equilibrated during peak metamorphic conditions. This coincides with the eclogite-facies mineral inclusions in the rims of the pyrite and is further evidence for contemporaneous pyrite mineral formation and Mg metasomatism that initiated during prograde and continued through to peak metamorphic conditions (*35*).

### Perspectives

This study provides evidence for the fluid-mediated transfer of ^34^S-enriched S from serpentinite into the adjacent metagabbro at temperatures below the stability limit of antigorite (~650°C and 2 GPa). Our results imply that the transfer of ^34^S-enriched S into subducted crustal materials does not require the production of fluids that carry oxidized sulfur species by serpentinite dehydration (*26*, *27*, *29*). If the latter scenario was the only pathway of mobilizing seawater-derived sulfate into the source region of arc magmas, then ^34^S-enriched S signatures in primitive arc magmas would be restricted to subduction zones with warm slab Moho geotherms where intraslab serpentinite breakdown occurs at subarc depths, as subduction zones with cold slab Moho geotherms are predicted to cross the antigorite dehydration at postarc depths. However, primitive arc magmas are enriched in ^34^S-enriched S compared to MORB, regardless of the slab thermal structure (*16*). This requires a universal transfer mechanism of sulfur that operates over the range of slab top geotherms, from cold to warm, across subduction zones. We argue that the fluid-mediated mass transfer between juxtaposed serpentinite and subducted crustal materials is a universal process that can lead to the formation of metasomatic rocks with geochemical and redox characteristics that are distinct from their protoliths. This fluid-mediated mass transfer was a consequence of the juxtaposition of chemically disparate rocks at high *P*-*T* conditions in water-rich environments, such as along the subduction interface or within intraslab shear zones. We predict that serpentinite-equilibrated fluids carry reduced S species at temperatures below 550°C at forearc and subarc depths. In the Voltri Massif, the fluid-mediated transfer of S involved simultaneous whole-rock oxidation of Fe, enrichment in fluid-mobile trace elements, and formation of pyrite with ^34^S values up to +12.5‰ in the reacted eclogitic metagabbro. Since the lithologies at the Voltri Massif represent exhumed materials from the slab-mantle interface relevant to subduction zones with intermediate to cold slab tops (*75*), we argue that similar fluid-mediated transfer between mafic and ultramafic rocks is likely pervasive in other subduction zones as suggested previously [e.g., (*76*, *77*)]. The process exemplified in the Voltri Massif accounts for the transfer of ^34^S-enriched S carried by subducted serpentinite and a subsequent enrichment of ^34^S in adjacent metacrustal or metasedimentary material upon fluid-mediated reaction at relatively low temperatures. This mechanism can act in concert with other proposed mechanisms that require fluid production during the breakdown of hydrous minerals in the slab at higher temperature conditions [e.g., (*6*, *27*, *67*)]. Once the fluid leaves the serpentinite-metagabbro transect, the fluid properties change depending on the fluid pathway toward the mantle. For example, if fluids traverse through metasedimentary layers that contain a lot of Mn oxides, then this would cause consequent oxidation of the fluid (*33*).

Our study provides insights into how ^34^S-enriched S can be transferred from subducted serpentinite into adjacent metagabbro by fluid-rock interactions. We envision that this process is likely pervasive in other subduction zones regardless of the slab geotherm and the thickness of the overriding crust. Subsequent melting of metasomatized, oxidized, and ^34^S-enriched metacrustal or metasedimentary rocks along the subduction interface or in mantle-wedge diapirs would produce melts that simultaneously display the elevated *f*o_2_ and S isotope signatures of arc magmas worldwide.

## MATERIALS AND METHODS

### Mineralogy and petrology

Sample preparation was done in the laboratories of the Freie Universität Berlin for the V18 series and at Virginia Tech for the V17 series samples (see data S1), which were initially processed to fine-grained gravel. Samples were cut into cubes using a rock saw, cleaned with acetone, ethanol, and deionized water to avoid cross-contamination and adherent rock dust, and then crushed to clay sized particles using an agate ball mill. In addition, sample blocks were cut for thin section preparation that was used for optical microscopy, electron microprobe analyses, and scanning electron microscopy.

Optical microscopy using reflected and transmitted light was conducted to identify silicate and sulfide mineral assemblages. The chemical composition of sulfide and silicate minerals was determined in selected samples on a JEOL JXA-8200 electron microprobe at the Freie Universität Berlin. Operating conditions for silicates were 15-kV acceleration voltage, 20-nA beam current, and 10 s per peak counting time. For sulfides, operating conditions were 20-kV acceleration voltage, 20-nA beam current, and 30-s counting time on the peak and 15 s on the background. The beam diameter was increased to 2 μm. The analytical reproducibility for the major elements is <0.5% for silicates, <0.2% for sulfides, and generally <4% for minor elements. For the sulfides, the detection limits were as follows: S < 0.009 wt %, Fe < 0.008 wt %, Ni < 0.008 wt %, Cu < 0.011 wt %, Co < 0.009 wt %, Zn < 0.014 wt %, As <0.045 wt %, and Sb < 0.015 wt %. Natural and synthetic mineral standards were used for calibration. Element distribution maps were carried out at 20 kV and 100 nA and at 50-ms dwell time.

### Bulk rock sulfur concentration and isotope analyses

Sulfur contents and isotope compositions were determined by extracting the sulfur from bulk rock powders using a slightly modified version of the methods described by Canfield *et al.* (*78*) and Tuttle *et al.* (*79*), which allow separate extraction of the acid volatile sulfide (AVS), chromium reducible sulfide (CRS), and sulfate fraction. Extraction proceeds in two steps: Initially, 15 to 20 g (depending on total sulfur contents) of bulk rock powder is reacted with 6 M HCl in an inert N_2_ atmosphere. This results in the release of H_2_S from AVS [e.g., pyrrhotite (Fe_1−*x*_S), pentlandite (Fe,Ni)_9_S_8_, and millerite (NiS)], which is transferred into a Zn-acetate trap solution to form ZnS. Tin(II) chlorite was added to the sample powder to reduce Fe^3+^ possibly present to Fe^2+^ and prevent oxidation of H_2_S to elemental sulfur. Sulfur bound in sulfates is recovered by reacting the HCl solution from the AVS extraction with BaCl_2_ to form BaSO_4_. In the second step, the remaining powder is reacted with a chromium(II)-chloride solution to dissolve CRS [e.g., pyrite (FeS_2_) and chalcopyrite (CuFeS_2_)], which is, similar to the first step, transferred as H_2_S into a Zn-acetate trap solution to form ZnS. Subsequently, the ZnS is converted to Ag_2_S through addition of AgNO_3_ to the Zn-acetate trap solution. The Ag_2_S precipitates were then filtered, and the BaSO_4_ was centrifuged. The amounts of collected AVS, CRS, and sulfate were determined gravimetrically and were corrected by the signal from the elemental analyzer. Detection limits for sulfate and sulfide concentrations depend on the amount of sample powder processed and were varied on the basis of prior mineralogical investigations; for 25 g of sample powder detection, limits are sulfate of 1 μg/g (corresponding to 0.0002 g of BaSO_4_ extract) and sulfide of 3 μg/g (corresponding to 0.0002 g of Ag_2_S extract).

Determination of the ^34^S/^32^S ratios was done on a Thermo Fisher Scientific MAT 253 isotope ratio mass spectrometer combined with a Eurovector elemental analyzer from HEKAtech at the Freie Universität Berlin. For the measurement, 400 to 800 μg of BaSO_4_ or Ag_2_S sample powder was weighted into 3.3-mm × 5-mm tin capsules, and divanadium pentoxide (V_2_O_5_) was added to enhance combustion. Isotope species of sulfur were measured in the form of SO_2_. Calculations of ^34^S/^32^S ratios were done with the external calibration method. For this, seven in-house K_2_SO_4_ standards were measured before each sample set. The ^34^S/^32^S ratios are expressed using the conventional δ^34^S notation using the Vienna Canyon Diablo Troilite (V-CDT). The V-CDT was calculated using an SO_2_ reference gas measured three times before each sample. To correct machine drift and memory effects on the resulting δ^34^S (V-CDT), the international standards IAEA S-1, S-2, and S-3 for Ag_2_S and IAEA SO-5 and SO-6 and NBS-127 for BaSO_4_ were measured in each run. The precision (1σ) of the measurements is ±0.03‰ for sulfides (IAEA S-2) and ±0.51‰ for sulfate (NBS-127). The analytical error of the mass spectrometer is lower than 2σ = 0.2‰.

### In situ isotope analyses

In situ analyses of sulfur isotope compositions of pyrite grains were determined on four selected metagabbro samples at increasing distance from the serpentinite contact. Pyrite grains were analyzed for δ^34^S (V-CDT; ^34^S/^32^S = 0.044163) (*80*) using a CAMECA IMS 1280-HR Secondary Ion Mass Spectrometer (SIMS) at the German Research Centre for Geosciences (GFZ) in Potsdam. Thin sections were cut into 25.4-mm-diameter disks. These were cleaned in high-purity ethanol ultrasonic bath and coated with 35-nm gold to provide electrical conductivity during analysis. A 2-nA Cs^+^ primary beam was focused to a 10-μm-diameter spot with a total impact energy of 20 keV. A normal incidence electron gun was used for charge compensation. Before data collection, each analytical site was sputtered for 100 s with a 20-μm raster to remove the gold coating, suppress any surface contaminants, and establish equilibrium sputtering conditions. Data collection used a 15-μm raster ensuring a flat-bottom crater geometry. Each analysis was preceded by automatic centering of the field aperture in *X* and *Y* and the contrast aperture in *X*. The field aperture was set to 4000 μm by 4000 μm (resulting in a field of view of 40 μm), and the contrast aperture had a diameter of 400 μm. The energy slit had a width of 40 eV, and it was mechanically positioned 5 V below maximum transmission at the start of the analytical sequence. Data were collected in multicollection mode using two Faraday cup detectors (L’2 for ^32^S^−^ and FC2 for ^34^S^−^) and a 80-μm-wide entrance slit. The exit slits for the L’2 and FC2 detectors were set to 500 and 250 μm in widths, respectively. Typical count rates on ^32^S in the Balmat pyrite reference material were on the order of 1.7 × 10^9^ to 2.6 × 10^9^ counts/s. The mass resolution was set at *M*/∆*M* ≈ 3800 (10% of peak height). Each analysis lasted ~4 min including presputtering, autocentering, and data acquisition routines, which consisted of 20 integrations of 4 s each. The instrumental mass fractionation and signal drift with time were monitored by repeated measurements throughout the analytical session in the Balmat pyrite reference material (δ^34^S_V-CDT_ = +15.1‰) (*81*) located in a different grain mount. No significant drift was observed, and the analytical session yielded a repeatability of ±0.12‰ (2 SD, 2 s). We estimate that the total analytical uncertainty on individual analyses is better than ±1.4‰ (2 s), where the main sources of analytical uncertainty are the reported heterogeneities and assigned uncertainties on the bulk characterization of the Balmat pyrite (*81*). Nabhan *et al.* (*82*) did not observe any systematic correlation between ^34^S/^32^S and the Co + Ni content of pyrite up to 5.5 wt %; hence, no correction for analytical bias related to chemical variation is required within this chemical range. This applies to the pyrite grains targeted in this study, which have Co of <4 wt %.

### Thermodynamic reaction path modeling

We performed thermodynamic reaction path calculations using the EQ3/6 software package (*63*) and the DEW Model (DEW_2019 version) (*64*, *65*, *83*) database to further assess the alteration history and concomitant mineralogical changes during a fluid-mediated reaction between juxtaposed serpentinite and metamorphosed oxide gabbro (metagabbro) at subduction-zone conditions guided by *P*-*T* constraints from equilibrium pseudo-section and garnet isopleth models from previous studies [e.g., (*35*, *40*). In the modeling setup, a fluid equilibrated with a serpentinite assemblage [i.e., antigorite + clinopyroxene (diopside) + magnetite + brucite] was allowed to react with a model sulfide-bearing gabbro at subduction zone conditions (500°C and 1.5 GPa) over a range of *f*/*r* mass ratios and *f*o_2_ (log *f*o_2_). Although brucite was not observed in our studied serpentinite, previous studies have suggested that brucite was originally present in these rocks but has later reacted with serpentine to produce olivine and water during the early stages of subduction (*84*–*86*). In a first modeling setup, an aqueous fluid was allowed to equilibrate with a serpentinite assemblage [i.e., antigorite + diopside clinopyroxene (Cpx) + magnetite + brucite] using EQ3 at specific *P*-*T*-*f*o_2_ conditions. The concentrations of dissolved Al and Ca in the starting fluid were set to 10^−4^ and 10^−3^ *m*, respectively. This is an iterative process where in every calculation, the predicted fluid composition is checked if it is saturated with the serpentinite assemblage only (i.e., antigorite + Cpx + magnetite + brucite). In cases where the predicted fluid composition is saturated with other minerals other than the desired serpentinite assemblage, the pH is slightly modified until the fluid becomes saturated with only the serpentinite assemblage. This simplification is needed to ensure that the model setup captures the condition where the fluid is in equilibrium with only serpentinite assemblage before it reacts with the metagabbro. We prescribed ~1000 μg/g dissolved S in the starting fluid based on the average whole-rock sulfur content of serpentinite from the northern Apennine (S_total_ = 939 ± 2390 μg/g) (*13*).

Once the fluid is saturated with only the desired serpentinite assemblage, the fluid is allowed to react with a model gabbro using EQ6. The bulk mineralogy of the modeled sulfide-bearing gabbro protolith [i.e., 41% plagioclase + ~45% clinopyroxene + 5% tremolite + 5% magnetite + 4% pyrite + C (100 μg/g)] is based on the mineralogy and modes of oxide gabbros reported in the Voltri region (*87*) and is consistent with the reported mineralogy and models of oceanic oxide gabbro from the Atlantis Massif (*88*). We implemented titration models that can be used to assess heterogeneous phase equilibria in an advective system (e.g., fluid reaction along a high permeability pathway such as lithologic boundaries) and in a diffusive system (e.g., fluid-mediated diffusive transfer at the boundary of a metagabbro and serpentinite, where the fluid composition is buffered by the rock) (*89*). The reaction-path model portrays a system that is initially fluid-dominated but then becomes increasingly rock-dominated as more gabbroic material is added. In this model setup, a fluid that is equilibrated with serpentinite is titrated with 1 kg of model gabbro under isobaric and isothermal conditions. The *f*/*r* mass ratio decreases with increasing reaction progress (ξ). Despite the simplifications and limitations in the modeling approach, the reaction-path models can simulate the evolving fluid-rock equilibria starting from the serpentinite toward the serpentinite-metagabbro contact and into the metagabbro interior with increasing ξ (equivalent to decreasing *f*/*r* ratios). This simplification allows one to model local fluid-rock equilibria as fluid travels from serpentinite into metagabbro. To evaluate the effect of oxygen fugacity on the sulfide stability and speciation of sulfur species in the fluid, we performed reaction-path modeling over a wide range of log *f*o_2_ conditions (−24.7 to −20.0) within the redox stability of magnetite, bounded by the magnetite-wüstite buffer and hematite-magnetite buffer. The starting *f*o_2_ condition, which is within the redox stability of magnetite, is set at the beginning where the aqueous fluid was allowed to equilibrate with a serpentinite assemblage in EQ3. Subsequently, the *f*o_2_ is allowed to vary upon the reaction between serpentinite-equilibrated fluids with metagabbro as controlled by heterogeneous phase equilibria in EQ6. The reaction-path model outputs the evolving fluid chemistry and the metasomatic minerals as a function of *f*/*r* ratio. Results of the reaction-path models were compared to the sequence of silicate and sulfide mineral assemblages from petrographic observations. The model input and results can be found in the Supplementary Materials.

### Isotope mixing and fractionation models

To explain the observed S isotope trends, isotope fractionation was modeled for open and closed system process and mixing of different S sources. Mixing processes were modeled, assuming that the initial metagabbro had a composition of S_sulfide_ = 1500 μg/g and δ^34^S_sulfide_ = 1.7‰ according to the average bulk rock composition of the coronitic metagabbro, which is similar to gabbros that experienced minor ocean floor alteration (*11*). We assume that these represent the initial gabbro compositions before metasomatism occurred. This coincides with the S isotope composition of pyrite cores in V18-A06 and V18-A08 that are mostly around δ^34^S = 0 to 2‰.

Mixing is calculated as

$$S_{\text{final},\text{WR}}=\left(1-f\right)\times \;H_{2}S_{\text{metasom}.}+S_{\text{gabbro}}$$

whereby metasomatic H_2_S of 3500 μg/g is incrementally added. This will produce the S_sulfide_ contents observed in the samples with the highest bulk rock S contents (see data S1). Accordingly, metasomatic H_2_S input decreases from fraction (*f*) = 0 toward *f* = 1, with *f* = 1 representing the S content and δ^34^S composition of the initial gabbro. In all scenarios that include reduction of oxidized sulfur species, we use the fractionation between SO_4_^2−^ and H_2_S. Sulfate (SO_4_^2−^)–to–sulfide (H_2_S) conversion is associated with an isotope enrichment factor (ε) of 20.2 to 10.1‰ at 300 to 550°C, respectively, whereas slightly smaller isotope fractionation takes place between SO_2_ to H_2_S (ε = 13.8 to 6.4‰ at 300 to 550°C, respectively) (*90*). Isotope fractionation during pyrite precipitation from H_2_S has an enrichment factor (ε) of 1.2‰ at 300°C, 0.9‰ at 400°C, and 0.6‰ at 550°C (*90*). For all calculations, we used a temperature of 400°C to account for the fact that metasomatism could already have started during prograde metamorphism before reaching peak *P*-*T* conditions [see (*35*)]. In addition, fractionation factors decrease with increasing temperatures; thus, the shown calculations represent maximum values.

Note that the metasomatic H_2_S is either derived directly from the serpentinite, assuming negligible amounts of isotope fractionation, e.g., only during pyrite precipitation as the fluid is infiltrating and reacting with the surrounding lithology in zone IVa. In this scenario, we assume that all H_2_S derived from serpentinite is entering the metasomatic zone (zones II and III), is entirely oxidized, and reprecipitates as pyrite in zones IVa and IVb. In the second scenario, H_2_S is produced by infiltration of oxidized sulfate (as SO_4_^2−^), e.g., as seawater-derived sulfate from either sediments or serpentinites, and is subsequently reduced, followed by precipitation as pyrite in zones IVa and IVb. Additional calculations and scenarios are shown in figs. S5 to S8.

## References

[1] K. A. Evans, The redox budget of subduction zones. Earth Sci. Rev. 113, 11–32 (2012).

[2] A. G. Tomkins, K. A. Evans, Separate zones of sulfate and sulfide release from subducted mafic oceanic crust. Earth Planet. Sci. Lett. 428, 73–83 (2015).

[3] J.-L. Li, E. M. Schwarzenbach, T. John, J. J. Ague, F. Huang, J. Gao, R. Klemd, M. J. Whitehouse, X. S. Wang, Uncovering and quantifying the subduction zone sulfur cycle from the slab perspective. Nat. Commun. 11, 514 (2020). 31980597 10.1038/s41467-019-14110-4 PMC6981181

[4] J. P. Richards, The oxidation state, and sulfur and Cu contents of arc magmas: Implications for metallogeny. Lithos 233, 27–45 (2015).

[5] D. Canfield, The evolution of the Earth surface sulfur reservoir. Am. J. Sci. 304, 839–861 (2004).

[6] J. B. Walters, A. M. Cruz-Uribe, H. R. Marschall, Sulfur loss from subducted altered oceanic crust and implications for mantle oxidation. Geochem. Perspect. Lett. 13, 36–41 (2020).

[7] J. B. Walters, A. M. Cruz-Uribe, H. R. Marschall, Isotopic compositions of sulfides in exhumed high-pressure terranes: Implications for sulfur cycling in subduction zones. Geochem. Geophys. Geosyst. 20, 3347–3374 (2019).

[8] A. G. Tomkins, Windows of metamorphic sulfur liberation in the crust: Implications for gold deposit genesis. Geochim. Cosmochim. Acta 74, 3246–3259 (2010).

[9] K. A. Evans, A. G. Tomkins, The relationship between subduction zone redox budget and arc magma fertility. Earth Planet. Sci. Lett. 308, 401–409 (2011).

[10] A. Rielli, A. G. Tomkins, O. Nebel, J. Brugger, B. Etschmann, R. Zhong, G. M. Yaxley, D. Paterson, Evidence of sub-arc mantle oxidation by sulphur and carbon. Geochem. Perspect. Lett. 3, 124–132 (2017).

[11] J. C. Alt, Sulfur isotopic profile through the oceanic crust: Sulfur mobility and seawater-crustal sulfur exchange during hydrothermal alteration. Geology 23, 585–588 (1995).

[12] J. C. Alt, J. W. Burdett, Sulfur in Pacific deep-sea sediments (Leg 129) and implications for cycling of sediment in subduction zones. Proc. ODP, Sci. Results 129, 283–294 (1992).

[13] J. C. Alt, E. M. Schwarzenbach, G. L. Früh-Green, W. C. Shanks III, S. M. Bernasconi, C. J. Garrido, L. Crispini, L. Gaggero, J. A. Padrón-Navarta, C. Marchesi, The role of serpentinites in cycling of carbon and sulfur: Seafloor serpentinization and subduction metamorphism. Lithos 178, 40–54 (2013).

[14] T. Plank, H. D. Holland, K. K. Turekian, 4.17 – The Chemical Composition of Subducting Sediments, in *Treatise on Geochemistry*, H. D. Holland, K. K. Turekian, Eds. (Elsevier, ed. 2, 2014), pp. 607–629.

[15] P. J. Wallace, M. Edmonds, The sulfur budget in magmas: Evidence from melt inclusions, submarine glasses, and volcanic gas emissions. Rev. Mineral. Geochem. 73, 215–246 (2011).

[16] M. J. Muth, P. J. Wallace, Slab-derived sulfate generates oxidized basaltic magmas in the southern Cascade arc (California, USA). Geology 49, 1177–1181 (2021).

[17] J. C. Alt, W. C. Shanks, M. C. Jackson, Cycling of sulfur in subduction zones: The geochemistry of sulfur in the Mariana Island Arc and back-arc trough. Earth Planet. Sci. Lett. 119, 477–494 (1993).

[18] J. C. M. de Hoog, B. E. Taylor, M. J. van Bergen, Sulfur isotope systematics of basaltic lavas from Indonesia: Implications for the sulfur cycle in subduction zones. Earth Planet. Sci. Lett. 189, 237–252 (2001).

[19] J. D. Woodhead, R. S. Harmon, D. G. Fraser, O, S, Sr, and Pb isotope variations in volcanic rocks from the Northern Mariana Islands: Implications for crustal recycling in intra-oceanic arcs. Earth Planet. Sci. Lett. 83, 39–52 (1987).

[20] B. Debret, M. A. Millet, M. L. Pons, P. Bouilhol, E. Inglis, H. Williams, Isotopic evidence for iron mobility during subduction. Geology 44, 215–218 (2016).

[21] I. S. E. Carmichael, The redox states of basic and silicic magmas: A reflection of their source regions? Contrib. Mineral. Petrol. 106, 129–141 (1991).

[22] K. A. Kelley, E. Cottrell, Water and the oxidation state of subduction zone magmas. Science 325, 605–607 (2009). 19644118 10.1126/science.1174156

[23] J. C. Alt, W. C. I. Shanks, Stable isotope compositions of serpentinite seamounts in the Mariana forearc: Serpentinization processes, fluid sources and sulfur metasomatism. Earth Planet. Sci. Lett. 242, 272–285 (2006).

[24] M. L. Pons, B. Debret, P. Bouilhol, A. Delacour, H. Williams, Zinc isotope evidence for sulfate-rich fluid transfer across subduction zones. Nat. Commun. 7, 8 (2016). 10.1038/ncomms13794 PMC5171646 27982033

[25] E. M. Schwarzenbach, M. J. Caddick, M. Petroff, B. C. Gill, E. H. G. Cooperdock, J. D. Barnes, Sulphur and carbon cycling in the subduction zone mélange. Sci. Rep. 8, 15517 (2018). 30341323 10.1038/s41598-018-33610-9 PMC6195527

[26] A. Bénard, K. Klimm, A. B. Woodland, R. J. Arculus, M. Wilke, R. E. Botcharnikov, N. Shimizu, O. Nebel, C. Rivard, D. A. Ionov, Oxidising agents in sub-arc mantle melts link slab devolatilisation and arc magmas. Nat. Commun. 9, 3500 (2018). 30158630 10.1038/s41467-018-05804-2 PMC6115406

[27] B. Debret, D. A. Sverjensky, Highly oxidising fluids generated during serpentinite breakdown in subduction zones. Sci. Rep. 7, 10351 (2017). 28871200 10.1038/s41598-017-09626-y PMC5583334

[28] I. J. Parkinson, R. J. Arculus, The redox state of subduction zones: Insights from arc-peridotites. Chem. Geol. 160, 409–423 (1999).

[29] F. Piccoli, J. Hermann, T. Pettke, J. A. D. Connolly, E. D. Kempf, J. F. Vieira Duarte, Subducting serpentinites release reduced, not oxidized, aqueous fluids. Sci. Rep. 9, 19573 (2019). 31862932 10.1038/s41598-019-55944-8 PMC6925189

[30] K. A. Evans, S. M. Reddy, A. G. Tomkins, R. J. Crossley, B. R. Frost, Effects of geodynamic setting on the redox state of fluids released by subducted mantle lithosphere. Lithos 278–281, 26–42 (2017).

[31] J.-L. Li, R. Klemd, G. F. Huang, J. J. Ague, J. Gao, Unravelling slab δ^34^S compositions from in-situ sulphide δ^34^S studies of high-pressure metamorphic rocks. Int. Geol. Rev. 63, 109–129 (2021).

[32] C.-T. A. Lee, M. Erdman, W. Yang, L. Ingram, E. J. Chin, D. J. DePaolo, Sulfur isotopic compositions of deep arc cumulates. Earth Planet. Sci. Lett. 500, 76–85 (2018).

[33] J. Ague, S. Tassara, M. E. Holycross, J.-L. Li, E. Cottrell, E. M. Schwarzenbach, C. Fassoulas, T. John, Slab-derived devolatilization fluids oxidized by subducted metasedimentary rocks. Nat. Geosci. 15, 320–326 (2022).

[34] S. Tumiati, G. Godard, S. Martin, N. Malaspina, S. Poli, Ultra-oxidized rocks in subduction mélanges? Decoupling between oxygen fugacity and oxygen availability in a Mn-rich metasomatic environment. Lithos 226, 116–130 (2015).

[35] E. A. Codillo, F. Klein, B. Dragovic, H. R. Marschall, E. Baxter, M. Scambelluri, E. Schwarzenbach, Fluid-mediated mass transfer between mafic and ultramafic rocks in subduction zones. Geochem. Geophys. Geosyst. 23, e2021GC010206 (2022).

[36] E. Rampone, G. B. Piccardo, “The ophiolite-oceanic lithosphere analogue: New insights from the Northern Apennines (Italy)” in *Ophiolite and Oceanic Crust: New Insights from Field Studies and Ocean Drilling Program*, J. Dick, E. Moores, D. Elthon, A. Nicolas, Eds. (Geological Society of America, 2000), vol. 349, pp. 21–34.

[37] E. Rampone, A. W. Hofmann, G. B. Piccardo, R. Vannucci, P. Bottazzi, L. Ottolini, Trace element and isotope geochemistry of depleted peridotites from an N-MORB type ophiolite (Internal Liguride, N Italy). Contrib. Mineral. Petrol. 123, 61–76 (1996).

[38] G. B. Piccardo, E. Rampone, R. Vannucci, F. Cimmion, Upper mantle evolution of ophiolitic peridotites from the northern Apennine: Petrological constraints to the geodynamic processes. Mem. Soc. Geol. It 48, 137–148 (1994).

[39] B. Messiga, M. Scambelluri, Retrograde *P*-*T*-*t* path for the Voltri Massif eclogites (Ligurian Alps, Italy): Some tectonic implications. J. Metam. Geol. 9, 93–109 (1991).

[40] P. G. Starr, K. S. Broadwell, B. Dragovic, M. Scambelluri, A. A. Haws, M. J. Caddick, A. J. Smye, E. F. Baxter, The subduction and exhumation history of the Voltri Ophiolite, Italy: Evaluating exhumation mechanisms for high-pressure metamorphic massifs. Lithos 376–377, 105767 (2020).

[41] L. Federico, L. Crispini, M. Scambelluri, G. Capponi, Different PT paths recorded in a tectonic mélange (Voltri Massif, NW Italy): Implications for the exhumation of HP rocks. Geodinamica Acta 20, 3–19 (2007).

[42] M. Scambelluri, E. Rampone, Mg-metasomatism of oceanic gabbros and its control on Ti-clinohumite formation during eclogitization. Contrib. Mineral. Petrol. 135, 1–17 (1999).

[43] W. Bach, J. C. Alt, Y. Niu, S. E. Humphris, J. Erzinger, H. J. B. Dick, The geochemical consequences of late-stage low-grade alteration of lower ocean crust at the SW Indian Ridge: Results from ODP Hole 735B (Leg 176). Geochim. Cosmochim. Acta 65, 3267–3287 (2001).

[44] S. E. Alford, J. C. Alt, W. C. Shanks III, Sulfur geochemistry and microbial sulfate reduction during low-temperature alteration of uplifted lower oceanic crust: Insights from ODP Hole 735B. Chem. Geol. 286, 185–195 (2011).

[45] M. Scambelluri, E. H. H. Strating, G. B. Piccardo, R. L. M. Vissers, E. Rampone, Alpine olivine- and titanian clinohumite-bearing assemblages in the Erro-Tobbio peridotite (Voltri Massif, NW Italy). J. Metam. Geol. 9, 79–91 (1991).

[46] E. Rampone, A. Romairone, W. Abouchami, G. B. Piccardo, A. W. Hofmann, Chronology, petrology and isotope geochemistry of the Erro-Tobbio peridotites (Ligurian Alps, Italy): Records of Late Palaeozoic lithospheric extension. J. Petrol. 46, 799–827 (2004).

[47] Y. Lagabrielle, M. Lemoine, Alpine, Corsican and Apennine ophiolites: The slow-spreading ridge model. C. R. Acad. Sci. Ser. II 325, 909–920 (1997).

[48] M. Marroni, G. Molli, A. Montanini, R. Tribuzio, The association of continental crust rocks with ophiolites in the Northern Apennines (Italy): Implications for the continent-ocean transition in the Western Tethys. Tectonophysics 292, 43–66 (1998).

[49] E. Le Breton, S. Brune, K. Ustaszewski, S. Zahirovic, M. Seton, R. D. Müller, Kinematics and extent of the Piemont–Liguria Basin–Implications for subduction processes in the Alps. Solid Earth 12, 885–913 (2021).

[50] E. M. Schwarzenbach, M. Harris, “Hydrothermal alteration of the oceanic lithosphere” in *Reference Module in Earth Systems and Environmental Sciences* (Elsevier, 2023); 10.1016/B978-0-323-99762-1.00016-4.

[51] J. C. Alt, W. C. Shanks III, L. Crispini, L. Gaggero, E. M. Schwarzenbach, G. L. Früh-Green, S. M. Bernasconi, Uptake of carbon and sulfur during seafloor serpentinization and the effects of subduction metamorphism in Ligurian peridotites. Chem. Geol. 322–323, 268–277 (2012).

[52] E. M. Schwarzenbach, thesis, ETH-Zürich, Zurich (2011).

[53] E. Cannaò, M. Scambelluri, S. Agostini, S. Tonarini, M. Godard, Linking serpentinite geochemistry with tectonic evolution at the subduction plate-interface: The Voltri Massif case study (Ligurian Western Alps, Italy). Geochim. Cosmochim. Acta 190, 115–133 (2016).

[54] J. Labidi, P. Cartigny, J. L. Birck, N. Assayag, J. J. Bourrand, Determination of multiple sulfur isotopes in glasses: A reappraisal of the MORB δ34S. Chem. Geol. 334, 189–198 (2012).

[55] J. C. Alt, D. A. H. Teagle, “Hydrothermal alteration and fluid fluxes in ophiolites and oceanic crust” in *Ophiolites and Oceanic Crust: New Insights from Field Studies and the Ocean Drilling Program*, Y. Dilek, E. M. Moores, D. Elthon, A. Nicolas, Eds. (Geological Society of America, 2000), pp. 273–282.

[56] C. G. C. Patten, I. K. Pitcairn, D. A. H. Teagle, M. Harris, Sulphide mineral evolution and metal mobility during alteration of the oceanic crust: Insights from ODP Hole 1256D. Geochim. Cosmochim. Acta 193, 132–159 (2016).

[57] J.-L. Li, E. M. Schwarzenbach, T. John, J. J. Ague, S. Tassara, J. Gao, B. A. Konecke, Subduction zone sulfur mobilization and redistribution by intraslab fluid–rock interaction. Geochim. Cosmochim. Acta 297, 40–64 (2021).

[58] W. Su, E. M. Schwarzenbach, L. Chen, Y. Li, T. John, J. Gao, F. Chen, X. Hu, Sulfur isotope compositions of pyrite from high-pressure metamorphic rocks and related veins (SW Tianshan, China): Implications for the sulfur cycle in subduction zones. Lithos 348–349, 105212 (2019).

[59] K. A. Evans, A. G. Tomkins, J. Cliff, M. L. Fiorentini, Insights into subduction zone sulfur recycling from isotopic analysis of eclogite-hosted sulfides. Chem. Geol. 365, 1–19 (2014).

[60] F. Giacometti, K. A. Evans, G. Rebay, J. Cliff, A. G. Tomkins, P. Rossetti, G. Vaggelli, D. T. Adams, Sulfur isotope evolution in sulfide ores from Western Alps: Assessing the influence of subduction-related metamorphism. Geochem. Geophys. Geosyst. 15, 3808–3829 (2014).

[61] J. B. Walters, A. M. Cruz-Uribe, H. R. Marschall, B. Boucher, The role of sulfides in the chalcophile and siderophile element budget of the subducted oceanic crust. Geochim. Cosmochim. Acta 304, 191–215 (2021).

[62] J.-L. Li, J. Gao, R. Klemd, T. John, X. S. Wang, Redox processes in subducting oceanic crust recorded by sulfide-bearing high-pressure rocks and veins (SW Tianshan, China). Contrib. Mineral. Petrol. 171, 72 (2016).

[63] T. J. Wolery, “EQ3NR, a computer program for geochemical aqueous speciation-solubility calculations: Theoretical manual, users guide, and related documentation (version 7.0); Part 3“ (UCRL-MA-110662-Pt.3, ON: DE93005827, TRN: 93:004658, Lawrence Livermore National Lab, 1992).

[64] F. Huang, D. A. Sverjensky, Extended Deep Earth Water Model for predicting major element mantle metasomatism. Geochim. Cosmochim. Acta 254, 192–230 (2019).

[65] D. A. Sverjensky, B. Harrison, D. Azzolini, Water in the deep Earth: The dielectric constant and the solubilities of quartz and corundum to 60kb and 1200°C. Geochim. Cosmochim. Acta 129, 125–145 (2014).

[66] P. Beaudry, D. A. Sverjensky, paper presented at the AGU Fall Meeting, San Francisco (V43E-0213), 14 December 2013.

[67] K. A. Evans, B. R. Frost, Deserpentinization in subduction zones as a source of oxidation in Arcs: A reality check. J. Petrol. 62, (2021).

[68] P. Ulmer, V. Trommsdorff, Serpentine stability to mantle depths and subduction-related magmatism. Science 268, 858–861 (1995). 17792181 10.1126/science.268.5212.858

[69] E. M. Schwarzenbach, B. C. Gill, E. Gazel, P. Madrigal, Sulfur and carbon geochemistry of the Santa Elena peridotites: Comparing oceanic and continental processes during peridotite alteration. Lithos 252–253, 92–108 (2016).

[70] R. J. Crossley, K. A. Evans, H. Jeon, M. R. Kilburn, Insights into sulfur cycling at subduction zones from in-situ isotopic analysis of sulfides in high-pressure serpentinites and ‘hybrid’ samples from Alpine Corsica. Chem. Geol. 493, 359–378 (2018).

[71] J. C. Alt, W. C. Shanks, W. Bach, H. Paulick, C. J. Garrido, G. Beaudoin, Hydrothermal alteration and microbial sulfate reduction in peridotite and gabbro exposed by detachment faulting at the Mid-Atlantic Ridge, 15° 20′ N (ODP Leg 209): A sulfur and oxygen isotope study. Geochem. Geophys. Geosyst. 8, 10.1029/2007GC0016 (2007).

[72] J. Liebmann, E. M. Schwarzenbach, G. L. Früh-Green, C. Boschi, S. Rouméjon, H. Strauss, U. Wiechert, T. John, Tracking water-rock interaction at the Atlantis Massif (MAR, 30°N) using sulfur geochemistry. Geochem. Geophys. Geosyst. 19, 4561–4583 (2018).

[73] E. Cannaò, M. Scambelluri, G. E. Bebout, S. Agostini, T. Pettke, M. Godard, L. Crispini, Ophicarbonate evolution from seafloor to subduction and implications for deep-Earth C cycling. Chem. Geol. 546, 119626 (2020).

[74] A. Kampschulte, H. Strauss, The sulfur isotopic evolution of Phanerozoic seawater based on the analysis of structurally substituted sulfate in carbonates. Chem. Geol. 204, 255–286 (2004).

[75] E. M. Syracuse, P. E. van Keken, G. A. Abers, The global range of subduction zone thermal models. Phys. Earth Planet. In. 183, 73–90 (2010).

[76] G. E. Bebout, Field-based evidence for devolatilization in subduction zones: Implications for arc magmatism. Science 251, 413–416 (1991). 17775106 10.1126/science.251.4992.413

[77] H. R. Marschall, J. C. Schumacher, Arc magmas sourced from mélange diapirs in subduction zones. Nat. Geosci. 5, 862–867 (2012).

[78] D. E. Canfield, R. Raiswell, J. T. Westrich, C. M. Reaves, R. A. Berner, The use of chromium reduction in the analysis of reduced inorganic sulfur in sediments and shales. Chem. Geol. 54, 149–155 (1986).

[79] M. L. Tuttle, M. B. Goldhaber, D. L. Williamson, An analytical scheme for determining forms of sulphur in oil shales and associated rocks. Talanta 33, 953–961 (1986). 18964237 10.1016/0039-9140(86)80234-x

[80] T. Ding, S. Valkiers, H. Kipphardt, P. De Bièvre, P. D. P. Taylor, R. Gonfiantini, R. Krouse, Calibrated sulfur isotope abundance ratios of three IAEA sulfur isotope reference materials and V-CDT with a reassessment of the atomic weight of sulfur. Geochim. Cosmochim. Acta 65, 2433–2437 (2001).

[81] D. E. Crowe, R. G. Vaughan, Characterization and use of isotopically homogeneous standards for in situ laser microprobe analysis of ^34^S/^32^S ratios. Am. Mineral. 81, 187–193 (1996).

[82] S. Nabhan, M. Wiedenbeck, R. Milke, C. Heubeck, Biogenic overgrowth on detrital pyrite in ca. 3.2 Ga Archean paleosols. Geology 44, 763–766 (2016).

[83] D. A. Sverjensky, Thermodynamic modelling of fluids from surficial to mantle conditions. J. Geol. Soc. London 176, 348–374 (2019).

[84] D. Peters, T. Pettke, T. John, M. Scambelluri, The role of brucite in water and element cycling during serpentinite subduction–Insights from Erro Tobbio (Liguria, Italy). Lithos 360–361, 105431 (2020).

[85] M. Scambelluri, O. Müntener, J. Hermann, G. B. Piccardo, V. Trommsdorff, Subduction of water into the mantle: History of an Alpine peridotite. Geology 23, 459–462 (1995).

[86] O. Plümper, T. John, Y. Y. Podladchikov, J. C. Vrijmoed, M. Scambelluri, Fluid escape from subduction zones controlled by channel-forming reactive porosity. Nat. Geosci. 10, 150–156 (2017).

[87] R. Tribuzio, B. Messiga, R. Vannucci, P. Bottazzi, Rare earth element redistribution during high-pressure–low-temperature metamorphism in ophiolitic Fe-gabbros (Liguria, northwestern Italy): Implications for light REE mobility in subduction zones. Geology 24, 711–714 (1996).

[88] M. Godard, S. Awaji, H. Hansen, E. Hellebrand, D. Brunelli, K. Johnson, T. Yamasaki, J. Maeda, M. Abratis, D. Christie, Y. Kato, C. Mariet, M. Rosner, Geochemistry of a long in-situ section of intrusive slow-spread oceanic lithosphere: Results from IODP Site U1309 (Atlantis Massif, 30°N Mid-Atlantic-Ridge). Earth Planet. Sci. Lett. 279, 110–122 (2009).

[89] W. Bach, F. Klein, The petrology of seafloor rodingites: Insights from geochemical reaction path modeling. Lithos 112, 103–117 (2009).

[90] H. Ohmoto, R. O. Rye, “Isotopes of sulphur and carbon” in *Geochemistry of Hydrothermal Ore Deposits* (Wiley, 1979), pp. 509–567.

[91] G. Vignaroli, F. Rossetti, M. Bouybaouene, H.-J. Massonne, T. Theye, C. Faccenna, R. Funiciello, A counter-clockwise P–T path for the Voltri Massif eclogites (Ligurian Alps, Italy). J. Metam. Geol. 23, 533–555 (2005).

[92] D. L. Whitney, B. W. Evans, Abbreviations for names of rock-forming minerals. Am. Mineral. 95, 185–187 (2010).

