# Supplementary material for: Mobilization of isotopically heavy sulfur during serpentinite subduction
Source: Sci Adv. 2024 Aug 7;10(32):eadn0641. doi: 10.1126/sciadv.adn0641 (PMC11305377; doi:10.1126/sciadv.adn0641)
Supplement: Supplementary file 1 — Figs. S1 to S8 Legends for data S1 to S3 [file sciadv.adn0641_sm.pdf]

Science Advances
AAAS

Supplementary Materials for

Mobilization of isotopically heavy sulfur during serpentinite subduction

Esther M. Schwarzenbach *et al.*

Corresponding author: Esther M. Schwarzenbach, esther.schwarzenbach@unifr.ch

Sci. Adv. **10**, eadn0641
DOI: 10.1126/sciadv.adn0641

The PDF file includes:

Figs. S1 to S8
Legends for data S1 to S3

Other Supplementary Material for this manuscript includes the following:

Data S1 to S3

1 Sulfide mineralogy and in situ $\delta^{34}S$ values in pyrite

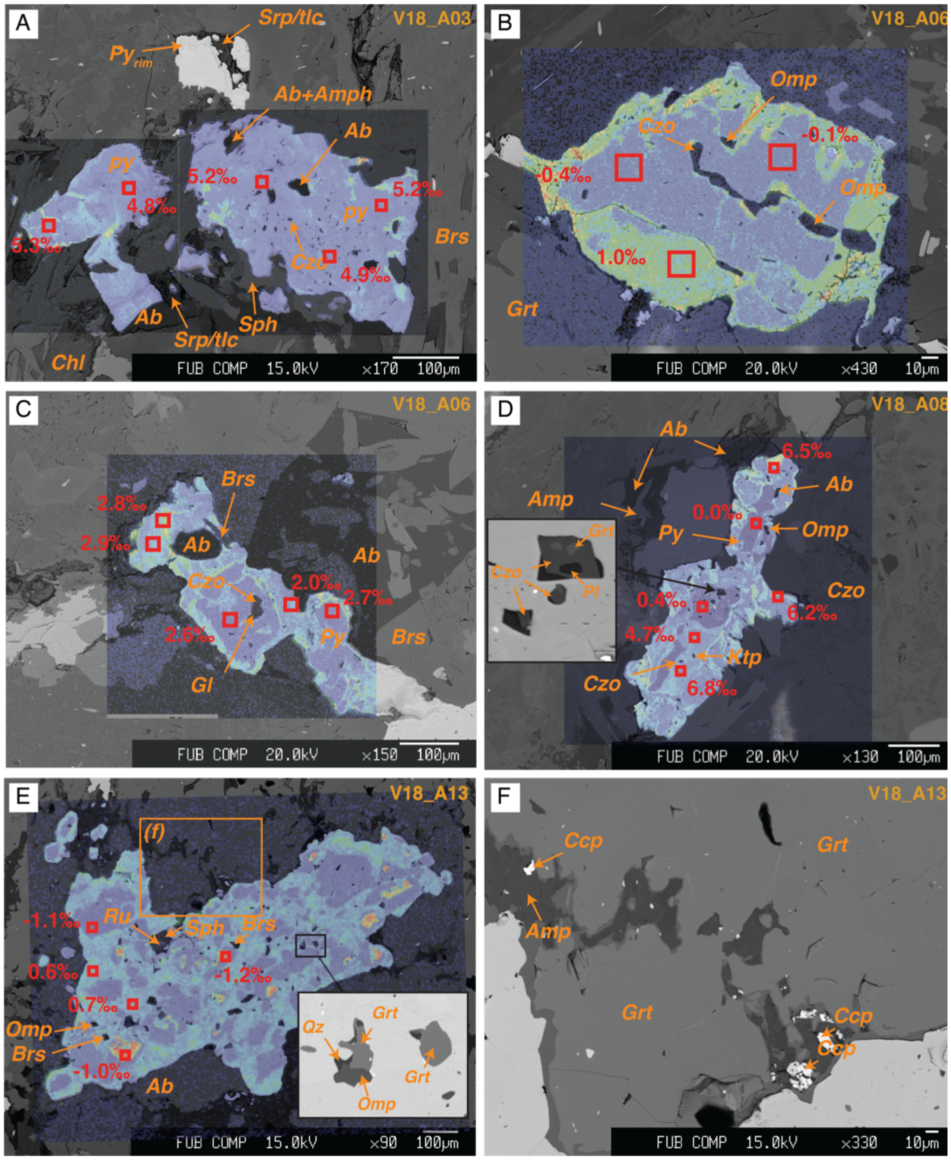

Fig. S-1.

BSE images showing the sulfide mineralogy of the studied samples with the Co element distribution overlain. Red squares indicate locations of SIMS pits and corresponding $\delta^{34}S$ values (in permil versus V-CDT). (A) Pyrite grains from zone IVa containing abundant silicate inclusions (amphibole, clinozoisite, albite) with some grains showing a distinctive, porous overgrowth rim (py_{rim}) that is in contact with a serpentine-talc matrix. (B) and (C) Pyrite grain from zone IVb that contains omphacite, glaucophane, and clinozoisite (epidote) inclusions. (D) Pyrite grain from zone IVb containing metamorphic minerals including omphacite, garnet, plagioclase and clinozoisite, (E) Pyrite containing numerous silicate inclusions (quartz + garnet + omphacite) next to garnet and (F) trace amount of chalcopyrite rimmed by amphibole. All silicate mineral inclusions are rimmed by pyrite with higher $\delta^{34}S$ values compared to the inner zones indicating that the ^{34}S-enriched pyrite formed during or subsequent to subduction metamorphism.

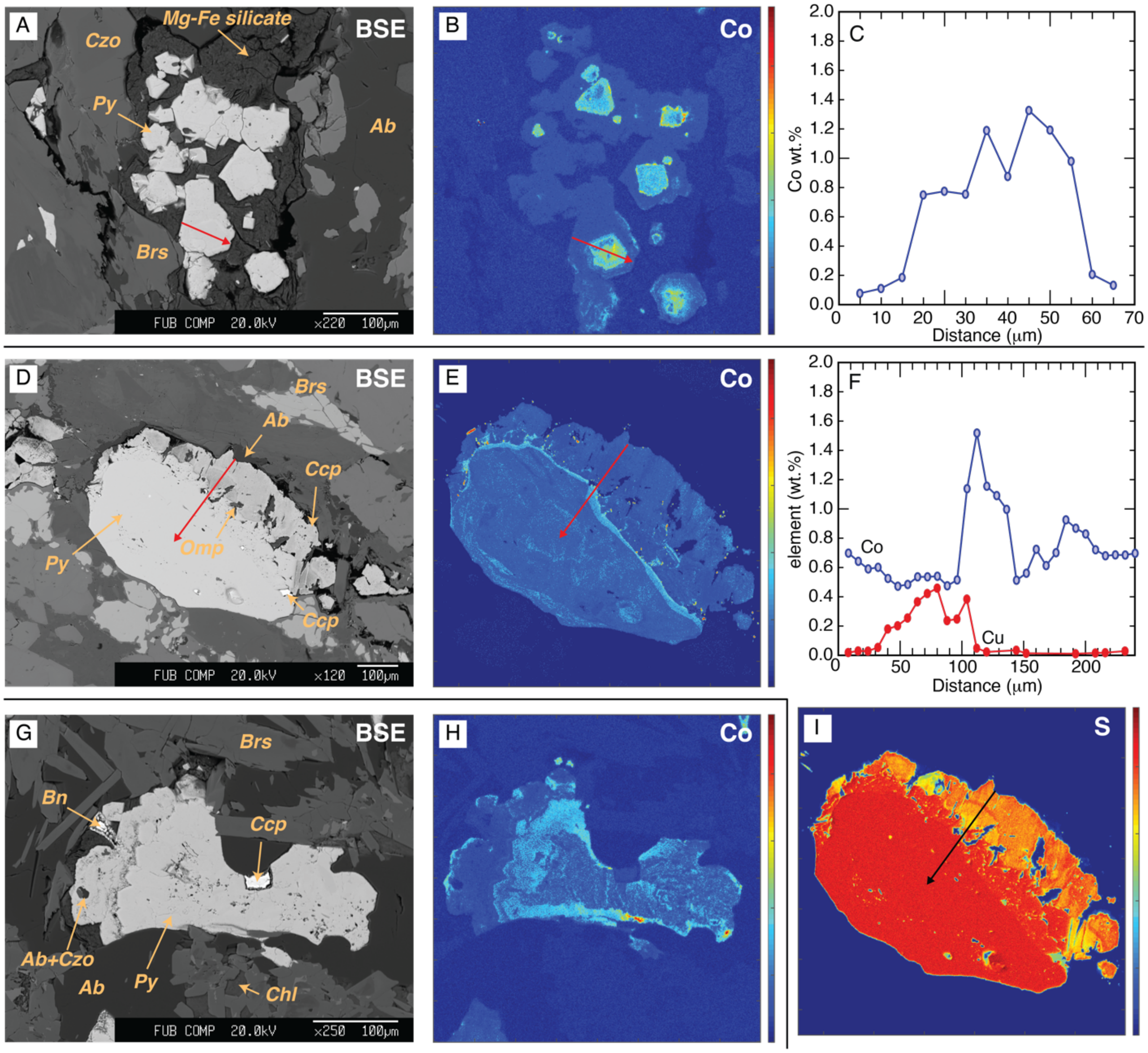

Fig. S-2.

BSE images and element distribution maps of sulfides from samples V18-A03 (zone IVa) and V18-A05 (zone IVb), as well as EMP travers analyses (in C and F) indicated by the red arrows. (A) to (C) Euhedral pyrite with a distinct Co zonation and high Co contents in the grain center. The pyrite is surrounded by a serpentine-talc matrix. (D) to (F), (i) Pyrite with corrosion rim that is characterized by lower S contents and considerable amounts of silicate inclusions (V18-A05). (g) to (h) Pyrite with a corrosion rim that is in contact with serpentine-talc intergrowths filling the surrounding, whereas the core shows an oscillatory zonation in Co (V18-A03).

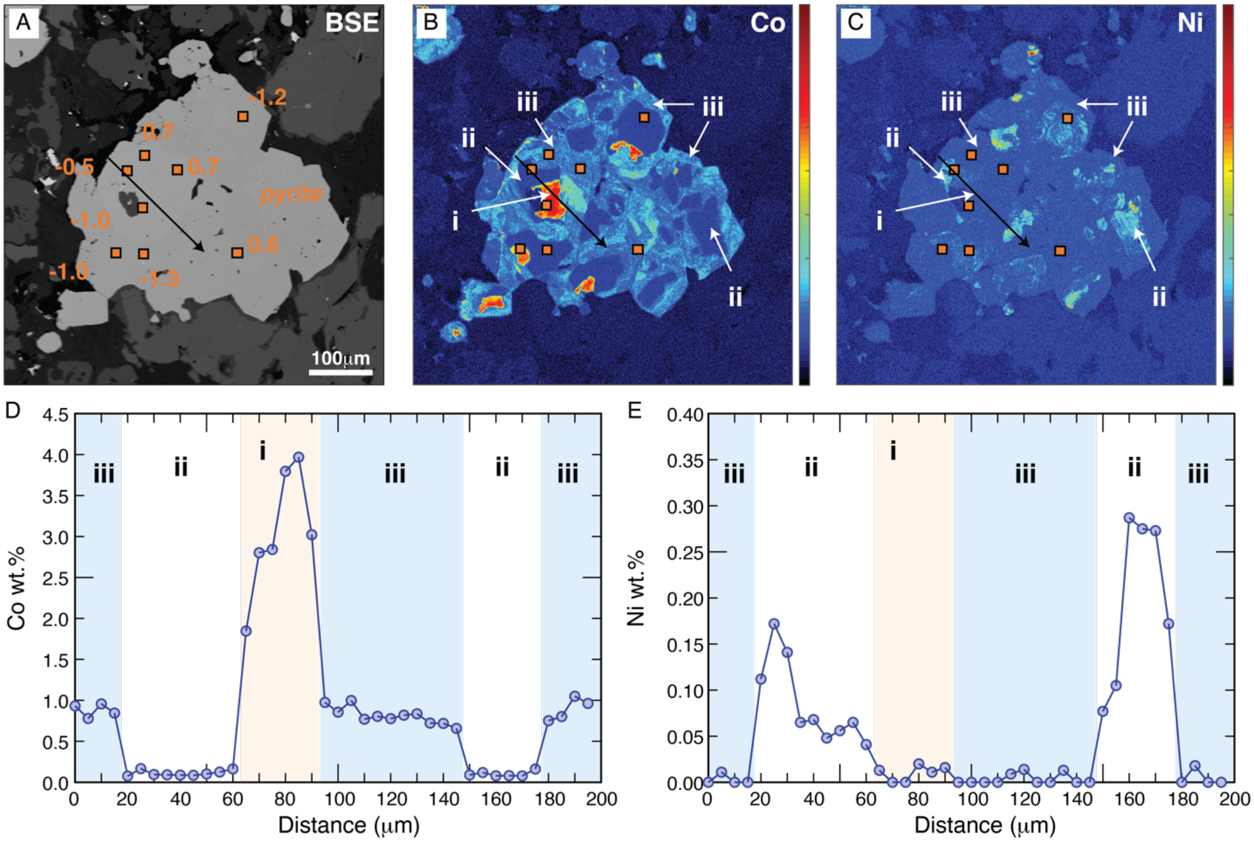

Fig. S-3.

BSE image (A) and element distribution maps of a pyrite grain from sample V18-A13 showing distinct zonations in Co concentrations (B) and slight variations in Ni contents (C). EMP traverse analyses (black arrow in (A), (B), (C)) allows identifying three zones based on distinct Co (D) and Ni (E) contents: i) an inner, Co-rich zone with up to 3.97 wt.% Co and very low Ni contents (<0.02wt%); ii) a very Co-poor zone (Co<0.2 wt%) with up to 0.7 wt.% Ni; and iii) an intermediate Co zone with Co contents around 0.7-0.9 wt.% and low Ni contents (<0.02wt%). Note that slight color variations in the BSE image suggests that several pyrite grains grew to one big grain during metasomatism. The *in situ* $\delta^{34}S$ values are shown in (A) in ‰ versus V-CDT and the location of the SIMS pits is shown in (A) to (C) by the orange squares.

2 Thermodynamic reaction path modelling

Thermodynamic reaction-path calculations were conducted with the EQ3/6 software package from (*63*) and the Deep Earth Water (DEW) Model (*64, 65, 83*) database.

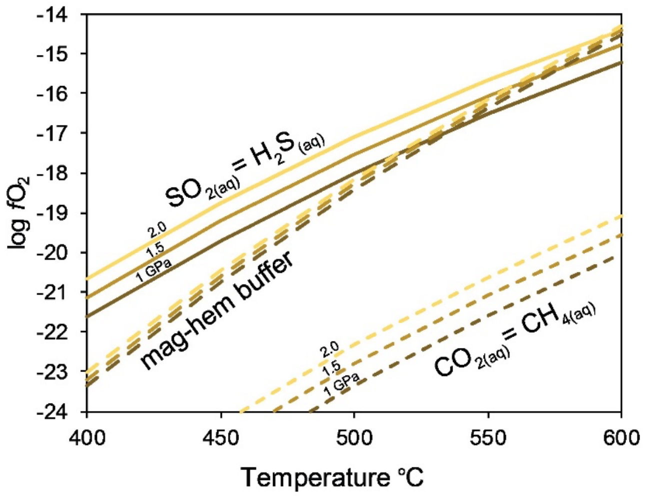

Fig. S-4.

Calculation of the $SO_{2(aq)} = H_2S_{(aq)}$ and $CO_{2(aq)} = CH_{4(aq)}$ equilibrium boundaries, and the magnetite-hematite buffer as a function of temperature (for pressures of 1.0, 1.5 and 2.0 GPa). This suggests that the more reduced species (H_2S) would coexist with magnetite-bearing serpentinite at the *P-T* conditions inferred for *HP* metamorphism and metasomatism of the Voltri Massif (see main text for discussion). The calculations were made using the DEW_2019 version.

3 Isotope fractionation calculations

Additional isotope fractionation and mixing models are plotted below. For details of the calculation see methods section in the manuscript. All mixing is calculated as:

$$S_{final,WR} = (1\text{-}f)^* \ H_2S_{metasom.} + S_{gabbro}$$

whereby metasomatic H_2S of 3500 µg/g is incrementally added to an eclogitic metagabbro with $S_{sulfide}$ =1500 µg/g and $\delta^{34}S_{sulfide}$ = 1.7‰. The metasomatic H_2S is either derived directly from the serpentinite or is produced by sulfate reduction either through open or closed system processes.

Different scenarios are shown below.

Fig. S-5 shows the effects of simple sulfur isotope mixing of H_2S (metasomatic H_2S) that is derived from serpentinite and is mixing with the eclogitic metagabbro during fluid infiltration and metasomatism (compare with Fig. 9C in the main text).

Fig. S-6 shows the effects of S isotope fractionation assuming open system fluid evolution in terms of sulfate availability, i.e., $\delta^{34}S_{metasom.\ H2S}$ is produced by the conversion $SO_4^{2-} \rightarrow H_2S$ and S isotope fractionation is only controlled by temperature and the value of the produced metasomatic H_2S does not change during reaction progress (no reservoir effects). This process would result in constant $\delta^{34}S$ values of the pyrite rims throughout the transect, however, open system processes are unlikely to take place in subduction zone settings.

Fig. S-7 shows the effects of S isotope fractionation following a closed system fluid evolution during sulfate reduction associated with fluid infiltration. In a closed system the conversion $SO_4^{2-} \rightarrow H_2S$ results in a continuous increase in $\delta^{34}S_{metasom.\ H2S}$ with $\delta^{34}S_{metasom.\ H2S,\ final} = \delta^{34}S_{sulfate,\ initial}$ (compare with Fig. 9D in the main text). This scenario would result in an increase in the $\delta^{34}S$ values of the pyrite rims along the transect into the metagabbro. In this case, the model fits most measured data using $\delta^{34}S$ sulfate values of ~15 to 22‰, with one data point requiring an initial $\delta^{34}S$ sulfate value of ~30‰.

Fig. S-8 shows the effects of S isotope fractionation following a Rayleigh distillation model. During Rayleigh distillation, the produced H_2S reaches highly positive $\delta^{34}S$ values as H_2S is continuously removed from the system during the conversion $SO_4^{2-} \rightarrow H_2S_{metasom.\ H2S}$. Note, the relatively constant $\delta^{34}S$ values of the pyrite rims throughout the transect and the lack of very high (i.e., >15‰) *in situ* $\delta^{34}S$ sulfide values point against such a process.

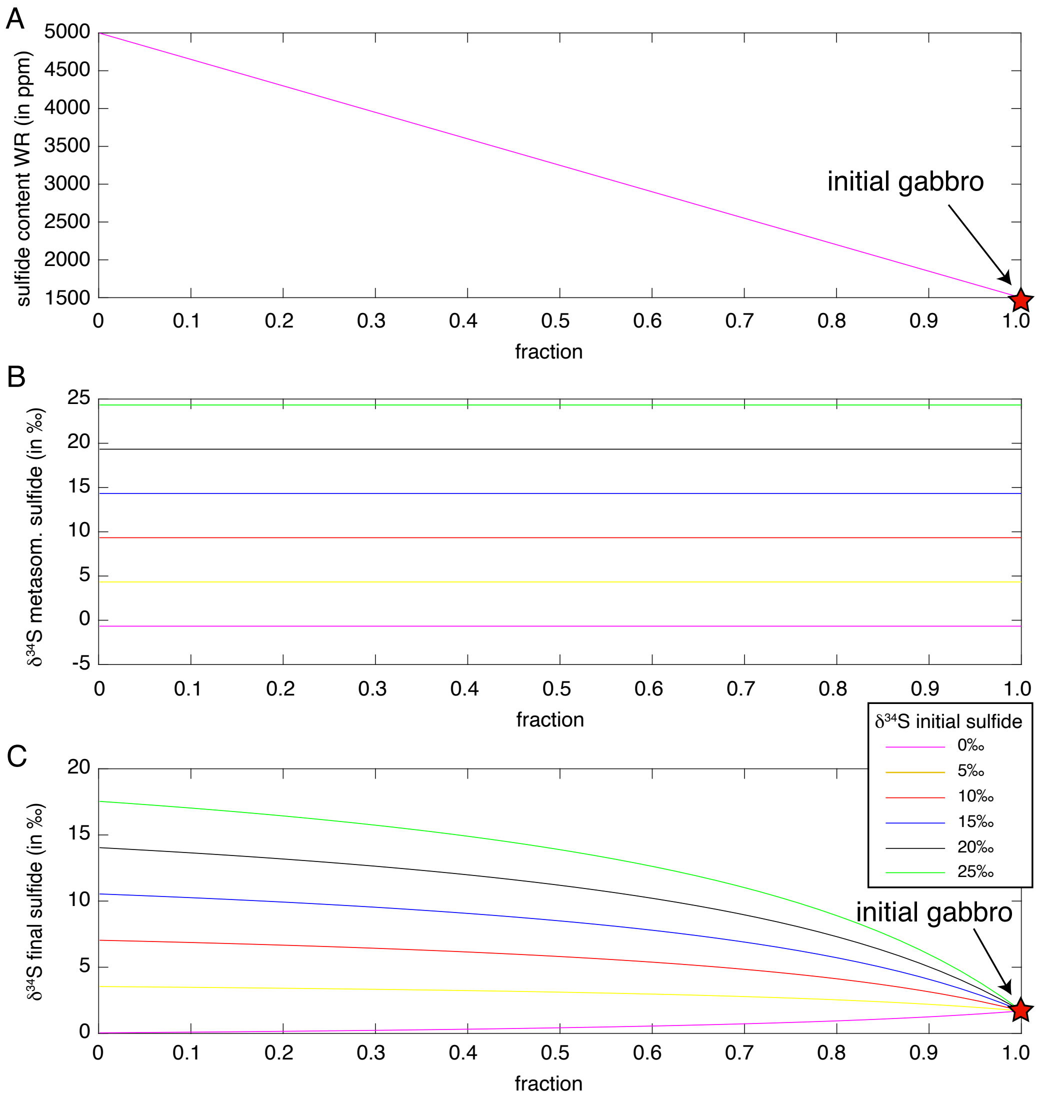

Fig. S-5.

Isotope mixing model of H_2S (metasomatic H_2S) influx from serpentinite and mixing with the eclogitic metagabbro (compare with Fig. 9C in the main text). Only isotope fractionation during pyrite precipitation from H_2S is considered, with $\varepsilon = 0.9$ at 400°C and 0.7‰ at 500°C. Initial sulfide content of the fluid is 3500 µg/g at variable initial $\delta^{34}S_{H2S}$, which is incrementally added to the gabbro ($S_{sulfide}$ = 1500 µg/g, $\delta^{34}S$= 1.7‰). Addition of metasomatic H_2S initiates from fraction (f) = 0 and decreases towards f = 1, with f = 1 representing the S content of the initial gabbro. (A) Sulfide content of the bulk rock, (B) evolution of the $\delta^{34}S$ value of the metasomatic H_2S assuming different initial $\delta^{34}S$ values between 0‰ and 25‰, (C) mixing of metasomatic H_2S assuming different initial $\delta^{34}S$ values between 0‰ and 25‰ with gabbro containing 1500 µg/g S and $\delta^{34}S$ = 1.7‰. All models were calculated at 500°C.

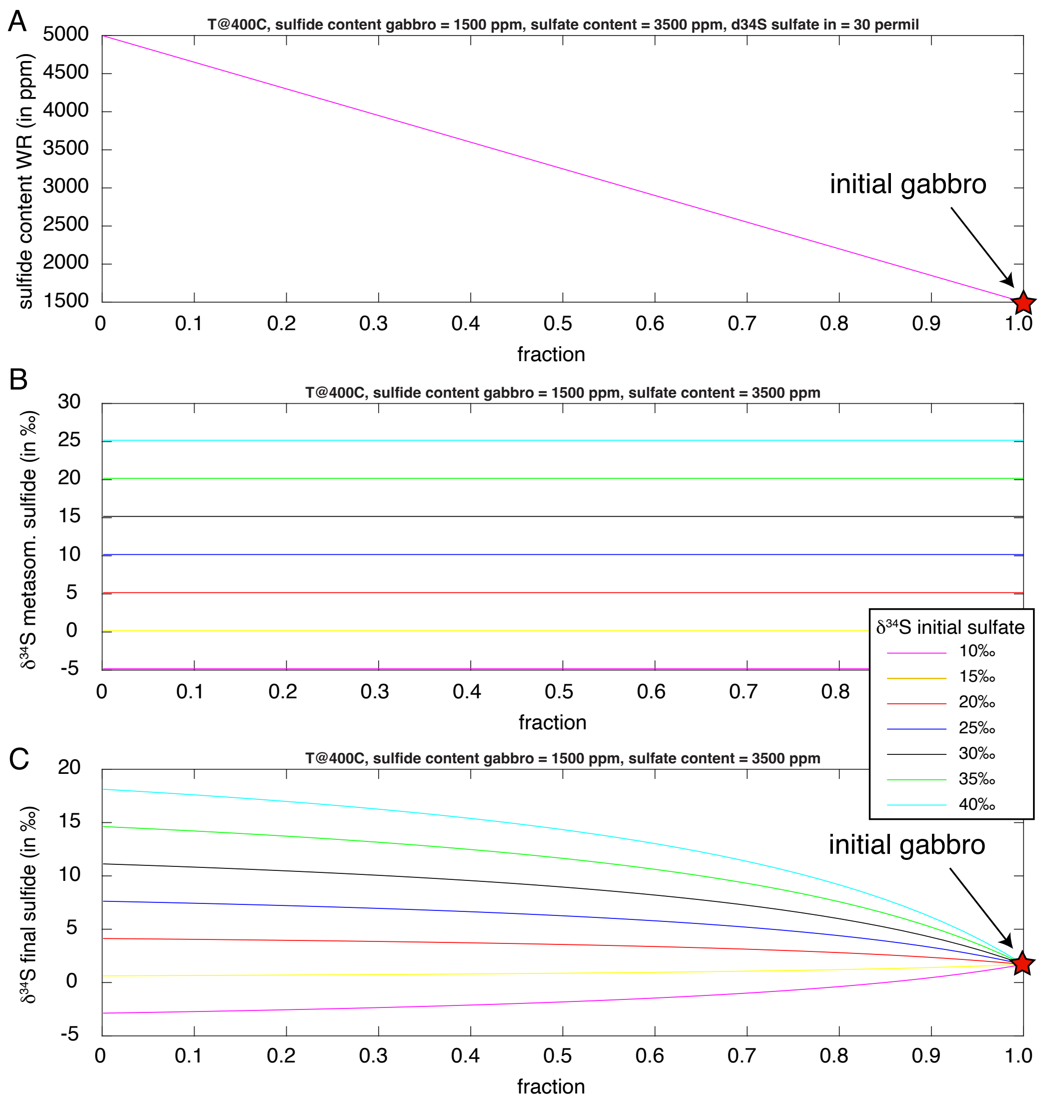

Fig. S-6.

Isotope fractionation model assuming open system fluid evolution in terms of sulfate availability, i.e., $\delta^{34}S_{metasom.\ H2S}$ is only controlled by temperature and does not change during reaction progress (no reservoir effects). The produced $H_2S_{metasom.}$, formed by sulfate reduction of 3500 µg/g sulfate, is incrementally added to the gabbro ($S_{sulfide}$ = 1500 µg/g, $\delta^{34}S$= 1.7‰). Addition of metasomatic H_2S initiates from fraction (f) = 0 and decreases towards f = 1, with f = 1 representing the S content of the initial gabbro. (A) Sulfide contents of the bulk rock, (B) evolution of the $\delta^{34}S$ value of the metasomatic H_2S assuming different initial $\delta^{34}S_{sulfate}$ values between 10‰ and 40‰, (C) mixing of metasomatic H_2S assuming different initial $\delta^{34}S_{sulfate}$ values between 10‰ and 40‰ with gabbro containing 1500 µg/g S and $\delta^{34}S$ = 1.7‰. All models were calculated at 400°C.

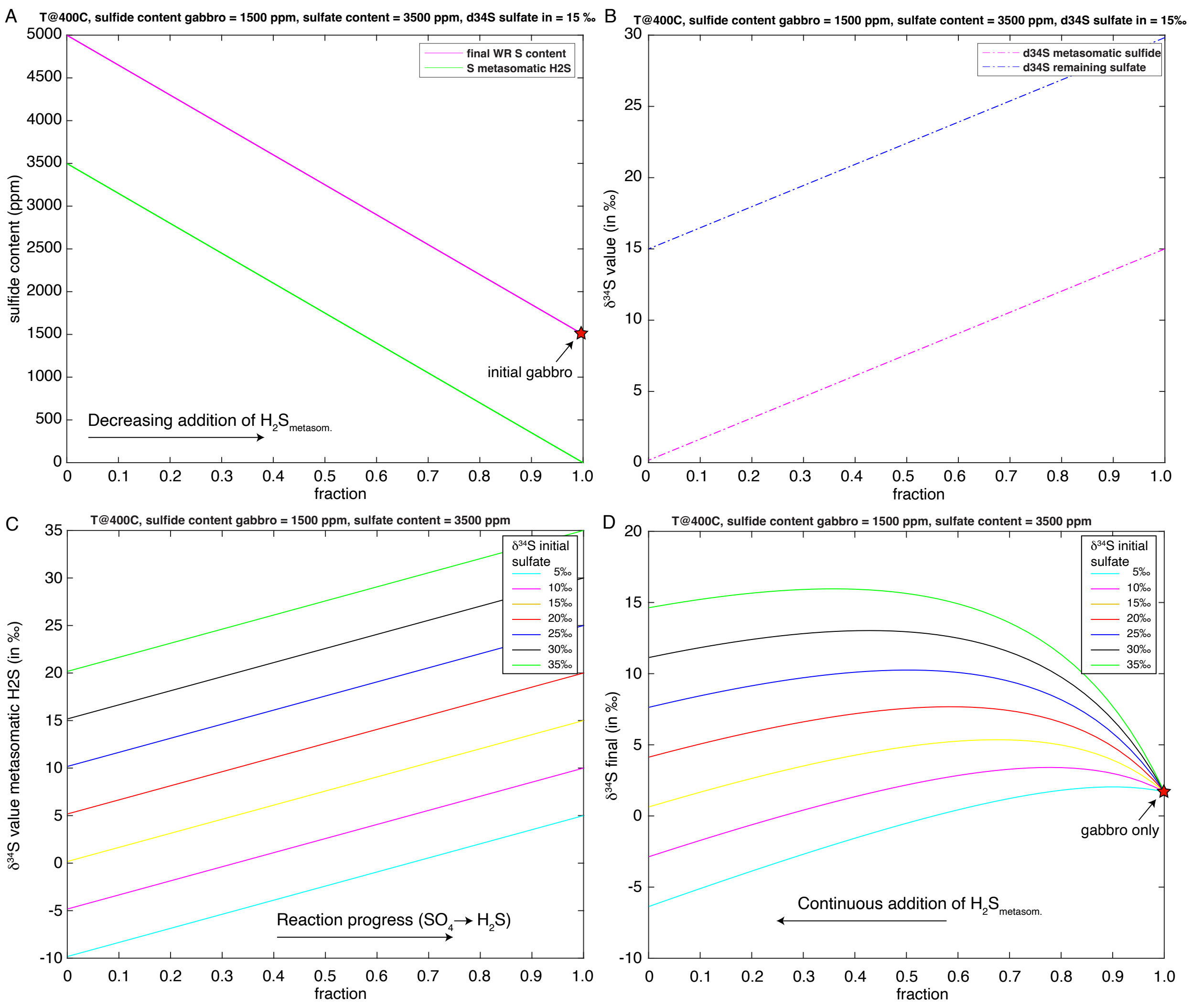

Fig. S-7.

Isotope fractionation model following a closed system fluid evolution, i.e., H_2S is produced by closed system sulfate reduction of 3500 µg/g sulfate, which results in a continuous increase in $\delta^{34}S_{\text{metasom. H2S}}$ with $\delta^{34}S_{\text{metasom. H2S, final}} = \delta^{34}S_{\text{sulfate, initial}}$ (compare with Fig. 9D in the main text). The produced $H_2S_{\text{metasom.}}$ is incrementally added to a gabbro with a S content of 1500 µg/g and $\delta^{34}S$ = 1.7‰. Addition of metasomatic H_2S initiates from fraction (f) = 0 and decreases towards f=1, with f = 1 representing the S content of the initial gabbro. (A) Sulfide contents of bulk rock and metasomatic H_2S as a function of the fraction, (B) evolution of the $\delta^{34}S$ values of the formed metasomatic H_2S and the remaining sulfate assuming an initial $\delta^{34}S_{\text{sulfate}}$ value of 15‰, (C) evolution of the $\delta^{34}S$ value of the metasomatic H_2S assuming different initial $\delta^{34}S_{\text{sulfate}}$ values from 5‰ to 35‰, (D) mixing of metasomatic H_2S assuming different initial $\delta^{34}S_{\text{sulfate}}$ values between 5‰ and 35‰ with gabbro containing 1500 µg/g S and $\delta^{34}S$ = 1.7‰. All models were calculated at 400°C.

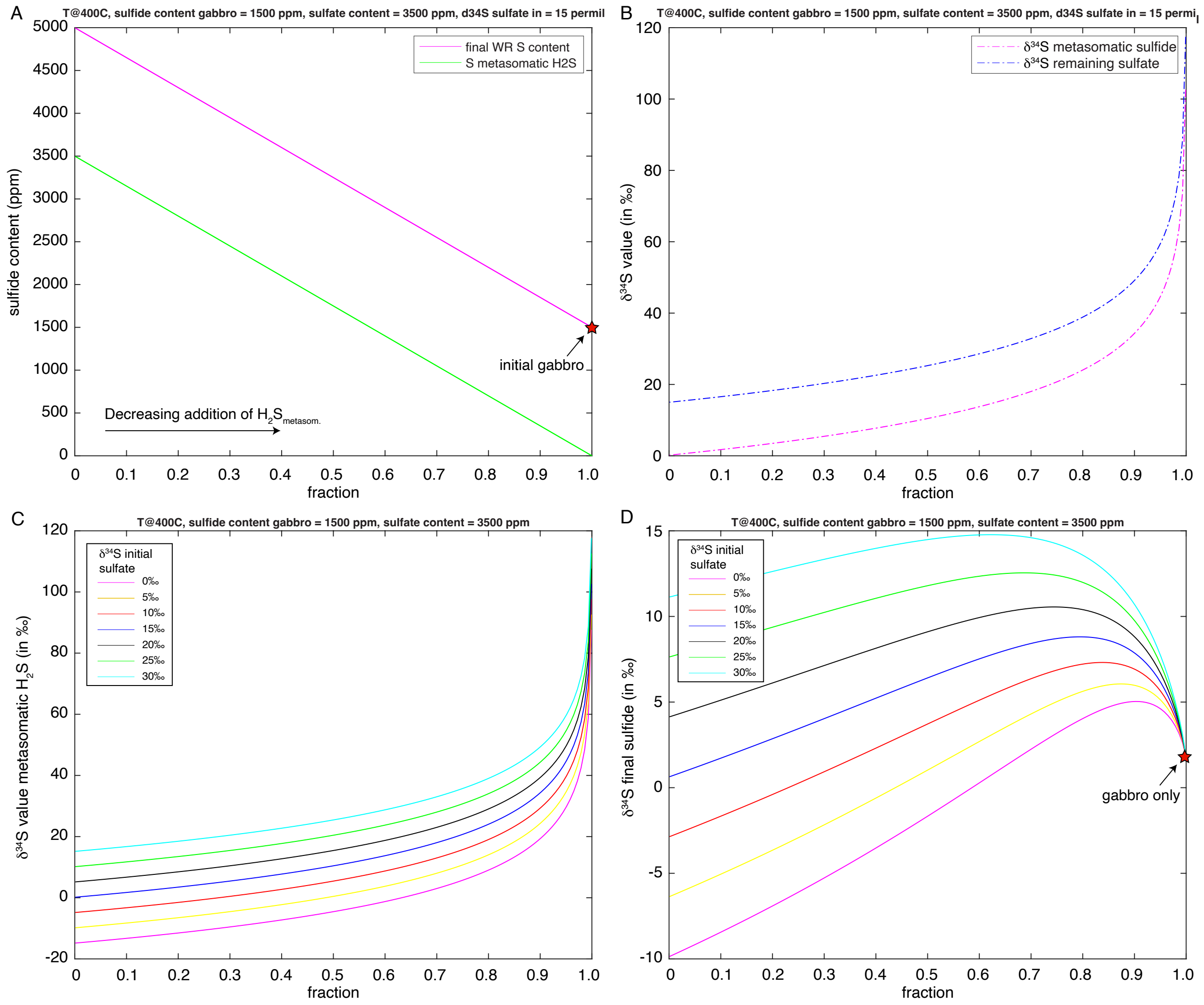

Fig. S-8.

Isotope fractionation model following a Rayleigh distillation model during sulfate reduction to form H_2S ($H_2S_{metasom.}$). The produced $H_2S_{metasom.}$ is incrementally added to a gabbro with a S content of 1500 µg/g and $\delta^{34}S = 1.7‰$. Addition of metasomatic H_2S initiates from fraction (f) = 0 and decreases towards f=1, with f = 1 representing the S content of the initial gabbro. (A) Sulfide contents of bulk rock and metasomatic H_2S as a function of the fraction, (B) evolution of the $\delta^{34}S$ values of the formed metasomatic H_2S and the remaining sulfate assuming an initial $\delta^{34}S_{sulfate}$ value of 15‰, (C) evolution of the $\delta^{34}S$ value of the metasomatic H_2S assuming different initial $\delta^{34}S_{sulfate}$ values from 0‰ to 30‰, (D) mixing of metasomatic H_2S assuming different initial $\delta^{34}S_{sulfate}$ values between 0‰ and 30‰ with gabbro containing 1500 µg/g S and $\delta^{34}S = 1.7‰$. All models were calculated at 400°C.

Data S-1. (separate file)
Sample list and sulfur geochemistry of the studied samples.

Data S-2. (separate file)
In-situ $\delta^{34}S$ values of pyrite measured by secondary ion mass spectrometry.

Data S-3. (separate file)
Sulfide mineral chemistry determined by electron microprobe analyses.
